# Spectral Representation of Neurochemicals With Phase, Frequency Offset, and Lineshape Invariance: Application to JPRESS for In Vivo Concentration and T_2_ Mapping by Deep Learning

**DOI:** 10.1002/mrm.70291

**Published:** 2026-02-07

**Authors:** Yan Zhang, Jun Shen

**Affiliations:** ^1^ National Institute of Mental Health, National Institutes of Health Bethesda Maryland USA

**Keywords:** deep learning, GRU, J‐point resolved spectroscopy (JPRESS), metabolite concentrations, T_2_, WaveNet

## Abstract

**Purpose:**

Using artificial intelligence neural networks to generate a representation that maps the input directly to neurochemical concentrations and metabolite‐level average transverse relaxation times (T_2_).

**Methods:**

The proposed model used time‐domain JPRESS data as input and was trained to be invariant to phase shifts, frequency offsets, and lineshape variations, using computer‐synthesized data without prior knowledge of in vivo metabolite concentration distributions. TE‐specific representations were generated using a combination of WaveNet and gated recurrent units (GRUs) and integrated into a unified JPRESS representation.

**Results:**

By focusing solely on target metabolite signals, the model effectively filtered out background signals, including spectral artifacts and unregistered metabolites. The predicted concentrations and metabolite‐level average T_2_ values were consistent with those reported in the literature. The model demonstrated robustness to phase shifts, frequency offsets, and line broadening. Additionally, it was capable of detecting low‐concentration neurochemicals, such as gamma‐aminobutyric acid (GABA), without spectral editing.

**Conclusion:**

This study demonstrates that deep learning can be used for automatically quantifying both metabolite concentrations and transverse relaxation times with high practical viability.

## 1 Introduction

Neurochemicals exhibit well‐defined spectral structures in magnetic resonance spectroscopy (MRS), which can be accurately simulated using quantum mechanics. These simulated spectra often serve as basis spectra for quantitative spectral fitting [1, 2, 3, 4, 5]. Alternatively, a dual‐task deep learning model has recently been proposed to predict in vivo metabolite concentrations at 3 Tesla without using spectral fitting [6]. Unlike traditional methods, this deep learning approach focuses solely on target metabolite signals, progressively learning their characteristics from local spectral features to the entire spectra, while filtering out unregistered signals, such as macromolecules and spectral artifacts, instead of learning them. It generates a latent representation that directly maps to target concentrations. In contrast, conventional spectral fitting methods require precise knowledge of all signals present in the input data to achieve accurate fits, which can be challenging in the presence of unknown or poorly defined signals in in vivo spectra, often caused by aberrant metabolic processes, scalp lipids, and macromolecules.

The dual‐task deep learning model is essentially an autoencoder, referred to as deepJPRESS hereafter. The latent representation learned by the encoder directly maps to the metabolite concentrations, while the decoder is simultaneously trained to reconstruct individual component free induction decay (FID) signals. During training, the network iteratively adjusts the weight parameters in both the encoder and the decoder to improve the accuracy of concentration prediction and individual FID reconstruction.

deepJPRESS uses time‐domain J‐point resolved spectroscopy (JPRESS) [7] data as input. Because metabolite concentrations directly correspond to free induction decay (FID) signal amplitudes, a time‐domain model can estimate concentrations without requiring explicit information about spectral lineshapes. This represents a major advantage over frequency‐domain approaches, in which spectral peaks must be quantitatively analyzed and lineshapes explicitly characterized. Accurately modeling in vivo spectral lineshapes is challenging due to experimental factors such as magnetic field inhomogeneity and eddy‐current–induced distortions [8, 9, 10, 11], which further complicate frequency‐domain analysis, whether performed by spectral fitting or deep learning.

The deepJPRESS decoder retains lineshape information by connecting to the inputs from lower‐level layers of the model. Learning to reconstruct individual FIDs allows the encoder to distinguish between different components and separate them within the latent representation space (dimensions = 128) [6]. The model employs WaveNet [12] to generate TE‐specific representations, which are then aggregated using gated recurrent unit (GRU [13]), creating a latent representation that directly maps to metabolite concentrations.

In this study, invariance to phase, frequency offset, and lineshape variations was introduced to the training of deepJPRESS, ensuring that the spectral representation remains unaffected by these confounding factors. To achieve lineshape invariance, a novel scheme termed pooling at the beginning (PAB) was devised. Leveraging the transverse relaxation (T_2_) information inherent in JPRESS data, the latent representation was utilized to map metabolite T_2_ values using artificial intelligence. Additionally, the study demonstrated that uncertainties in the predicted concentrations can be assessed using resampled datasets derived from the original test data and deepJPRESS predictions. Furthermore, a comparison between deepJPRESS and the traditional spectral fitting method, LCModel, demonstrated the superior performance of deepJPRESS.

## 2 Methods

### 2.1 Deep Learning Model

**FIGURE 1:**
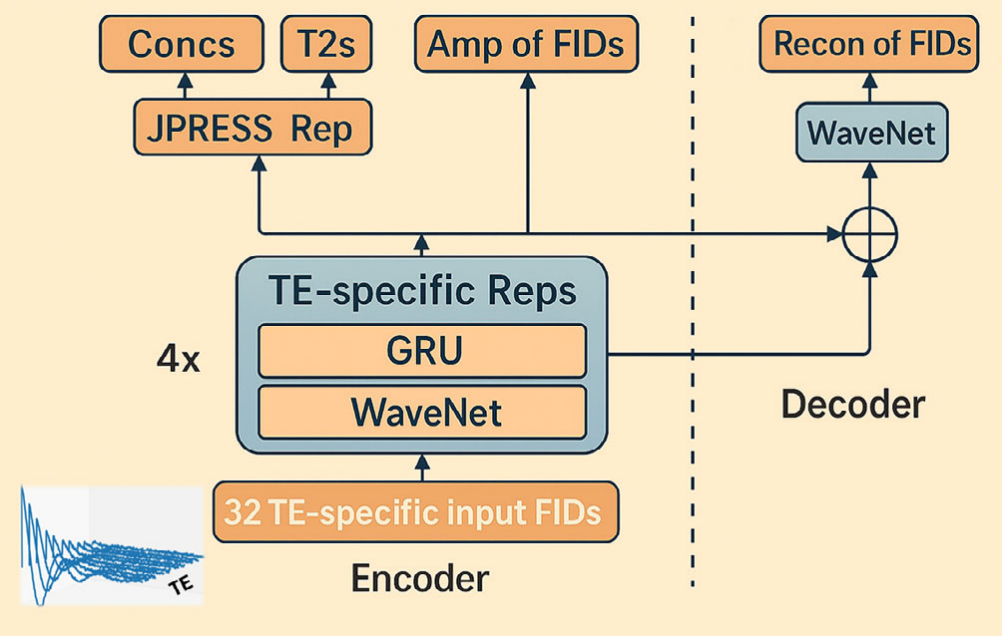
deepJPRESS takes a series of JPRESS time‐domain signals as input. Thirty two free induction decay (FID) signals with different TEs are arranged in ascending order, each consisting of 2048 complex data points, resulting in an input data format of (32, 2048, 2). The WaveNet block extracts spectral features from the target FIDs, and a gated recurrent unit (GRU) fuses the spectral features from different TE data, generating TE‐specific representations after applying pooling at the beginning (PAB). This procedure is repeated four times, forming the encoder shown on the left of the dotted line. Subsequently, global average pooling on the TE‐specific representations yields the JPRESS representation, which maps the input to metabolite concentrations and T_2_s. The TE‐specific representations are also used to predict the first‐point amplitudes of each FID for all target components. On the right is the decoder, which receives inputs from all the layers of the encoder to reconstruct individual component FIDs for all 32 TEs. Amp, amplitude; Concs, concentrations; Recon, reconstruction; Rep, representation.

The outline of the deepJPRESS model architecture is shown in Figure 1 (also see Script S1 for its TensorFlow implementation). TE‐specific representations are generated by a combined use of WaveNet and GRU before being aggregated to create the JPRESS representation. This representation serves as the output of the encoder and maps the input FIDs to metabolite concentrations and T_2_s.

The WaveNet block is a convolutional network that extracts metabolite spectral features. It uses dilated convolutions, rather than strides, to capture long‐range dependencies while keeping the input size unchanged. This structure was adopted because the TE‐specific representations needed to be iteratively processed to fuse spectral features of individual spectra through the GRU block.

An auxiliary output is included to forecast the amplitudes of individual FIDs, facilitating learning the JPRESS representation. The decoder utilizes outputs from multiple encoder layers to reconstruct target component FIDs.

### 2.2 Phase, Frequency Offset, and Lineshape Invariance

The deepJPRESS model was trained with a focus on achieving invariance with respect to phase shift, frequency offset, and lineshape (collectively referred to as PFL) variations, allowing the JPRESS representation to remain independent of these factors. To achieve this, the metabolite FIDs of training data were created with randomized phase shifts (0° to 360°), frequency offsets (−5 to 5 Hz relative to water frequency), and were then multiplied by a modified Voigt lineshape function:

(1) $$\text{lineshape}=\text{Voigt}\times \left(1-b_{1}t-b_{2}t^{2}-b_{3}t^{3}\right),$$

where the Lorentzian and Gaussian terms in the Voigt function for metabolite components ranged from π s^−1^ to 8π s^−1^ and from 9 to 21 s^−2^, respectively; the parameters *b*_1_, *b*_2_, and *b*_3_ were varied from −1.5 to 3.0 s^−1^, −3.0 to 30 s^−2^, and 0 to 210 s^−3^, respectively. The encoder was trained without incorporating the PFL factors. Random phase shifts and frequency offsets were independently applied across the 32 TE‐specific FIDs to ensure robustness against frequency drifts and phase variations occurring across individual echoes during prolonged in vivo data acquisition. In contrast, all 32 FIDs shared the same lineshape. The water PFL varied independently from that of the metabolites, preventing the model decoder from leveraging water PFL information. It is important to note that the decoder must recover the original PFL from the encoder in order to accurately reconstruct the individual component FIDs.

To evaluate the robustness of the pretrained deepJPRESS model to variations in PFL, test results were compared before and after applying PFL perturbations to the in vivo test datasets. For phase and frequency perturbations, a random phase shift between −180° and 180° and a random frequency offset between −5 and 5 Hz were independently applied to each TE‐specific FID across all datasets. For lineshape perturbation, a Voigt lineshape was randomly applied to the in vivo test data, with Lorentzian broadening parameters ranging from 0 to 5π s^−1^ and Gaussian broadening parameters from 0 to 15.5 s^−2^. The effectiveness of the PFL invariance was further confirmed by datasets derived from an individual in vivo data sample to avoid influences from inter‐sample variability.

### 2.3 PAB

**FIGURE 2:**
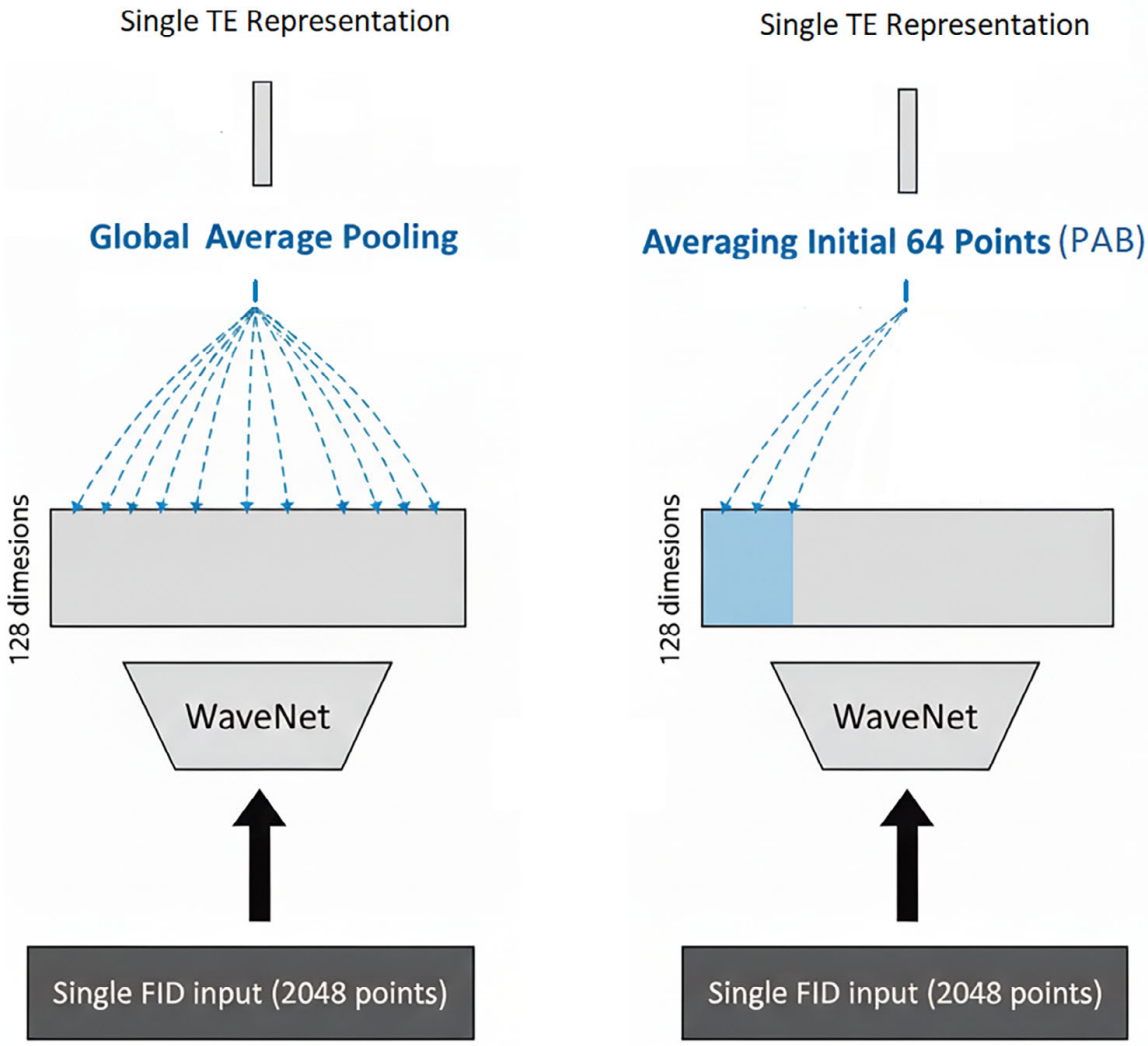
Comparison of conventional global average pooling (left panel) and the newly proposed pooling at the beginning (PAB) (right panel). In both cases, the WaveNet extracts spectral features from input free induction decay (FID) signals (2 channels, 2048 sampling points), generating a representation for each point with 128 dimensions. While global average pooling averages over the entire 2048 points, PAB achieves pooling using only the initial portion of the 2048 points.

TE‐specific representations were previously generated using a WaveNet neural network, followed by global average pooling over the 2048 sampling points of the FIDs [6], effectively summing all the points of the FIDs. In this study, a new strategy, pooling at the beginning (PAB), is proposed wherein only the initial 64 points are pooled to mitigate the influence of lineshapes on the spectral representations. Figure 2 illustrates the difference between global pooling and PAB strategies.

While ideally, the first point of each FID can be used to create the desired representations, this choice requires the WaveNet block to have sufficient dilation depths to capture feature correlations from long temporal distances. Two potential issues arise with the first‐point approach: (1) the first point of an in vivo FID signal could be corrupted due to hardware imperfections, built‐in digital filtering, and eddy currents, and (2) learning long‐distance temporal correlations can lead to overfitting of the training data. A common symptom of this overfitting is that the improved accuracy on the training dataset results in poorer performance on in vivo tests. In this study, the dilation depth was optimally set at 8, meaning that the longest hopping distance for the convolutions is 256 points, and, as a result, pooling on the initial 64 points was adopted for PAB. This PAB window was determined empirically by minimizing the training validation loss.

### 2.4 Metabolite‐Level Average T_2_ Labeling

Since the observed MRS signals are additive, representing the sum of all individual component contributions to the spectrum, for a metabolite consisting of *n* T_2_ subgroups, its FID signal can be expressed as:

(2) $$s\left(t\right)=\sum_{k=1}^{n}a_{k}\left(t\right)e^{-\frac{t}{T_{2}^{k}}},$$

where $a_{k}\left(t\right)$ denotes the subgroup *k* signal intensity in the absence of the transverse relaxation attenuation and $T_{2}^{k}$ is the T_2_ for the subgroup *k*. In the computer‐synthesized training dataset, a subgroup's T_2_ is altered by a pair of randomly varying terms, $\delta_{1}$ and $\delta_{2}$ as follows:

(3) $$\frac{1}{T_{2}^{k}}=\frac{1}{\text{preset}\;T_{2}^{k}}+\delta_{1}+\delta_{2}.$$

The resultant metabolite‐level average T_2_ target is given by $\left(\sum_{k=1}^{n}T_{2}^{k}\right)/n$. Due to the hierarchical nature of deep learning, the model first learns to characterize subgroups as local spectral features. Then, it extends this learning to the entire metabolite component; in doing so, the model predicts the average T_2_ for the metabolite. All metabolite subgroups shared the same *δ*_1_, which varies within a uniform distribution from −1.3 to 4.4, while water has a different *δ*_1_ in the range of 1.3 to 12.6. Each metabolite and its subgroups have an independent random *δ*_2_ varying from −0.63 to 0.63. Using Equation (3), the T_2_ values of a metabolite subgroup and water vary approximately within 90 to 590 ms and 75 to 1400 ms, respectively. The high upper limit of 1400 ms for water T_2_ was chosen to account for cases with dominant cerebrospinal fluid (CSF) contribution. The combined use of $\delta_{1}$and $\delta_{2}$ in Equation (3) enables all metabolites to experience the same global T_2_ variations while maintaining individual differences.

### 2.5 Training Datasets

The training datasets were generated following the same procedure as in the previous study [6]. The process can be summarized using the following pipeline: simulated FIDs → assignment of random concentrations and T_2_s → application of variations in phase, frequency offsets, and lineshape → injection of noise and extraneous peaks.

Fourteen metabolites were included: N‐acetylaspartate (NAA), N‐acetylaspartylglutamate (NAAG), creatine (Cr), phosphocreatine (PCr), phosphocholine (PCho), glycerophosphocholine (GPCho), glutamate (Glu), glutamine (Gln), glutathione (GSH), taurine (Tau), aspartate (Asp), myo‐inositol (mI), gamma‐aminobutyric acid (GABA), and lactate (Lac). NAA and NAAG, Cr and PCr, and PCho and GPCho were each combined into single components referred to as tNAA, tCr, and tCho, respectively, resulting in 12 targets (including water).

For the current study, an additional random frequency offset ranging from −1 to 1 Hz was applied to all chemical shifts of individual target metabolites when generating the training dataset (note that the frequency offset referred to here differs from that of PFL; the latter represents the frequency offset of the metabolites as a whole relative to water). To ensure generalizability and robustness for in vivo applications, the training data were created without a fixed PFL relationship between water and metabolites, and individual TE spectra had independent phase shifts and frequency offsets while sharing a lineshape described by Equation (1).

A total of 100 000 samples were generated with randomly assigned concentrations ranging from 0 to 20 mM. The median values of the uniformly distributed concentrations were as follows: NAA, 10 mM; NAAG, 5 mM; Cr, 5 mM; PCr, 5 mM; PCho, 5 mM; GPCho, 5 mM; Glu, 10 mM; Gln, 10 mM; GSH, 10 mM; Tau, 10 mM; Asp, 10 mM; mI, 10 mM; GABA, 10 mM; and Lac, 10 mM.

### 2.6 In Vivo Test Datasets

In vivo tests were conducted on 30 data samples described previously [6, 14]. All procedures were approved by the local Institutional Review Board (Protocol number: NCT01266577). Written informed consent was obtained from all participants. The region of interest was prescribed in the anterior cingulate cortex (ACC) with a voxel (2.0 × 2.0 × 4.5 cm^3^) dominated by gray matter (see Table S1 for additional details). A bi‐exponential fit was applied to unsuppressed water reference to remove CSF and to extrapolate the tissue water amplitude to TE = 0 [15]. The spectral data were subsequently normalized with respect to the tissue water amplitude. Spectral phase was corrected using the water reference to enable data visualization in the frequency domain.

### 2.7 Resampling‐Based Sensitivity Intervals

To evaluate the uncertainty of the predicted metabolite concentrations, we employed a resampling‐based approach to generate test datasets. Each test dataset was constructed by scaling the predicted concentrations using samples drawn from prior scaling distributions, with the condition that the target metabolite concentration remained fixed at the predicted value (i.e., scaled by 1). All predicted individual component FIDs, including the background signal, were then scaled accordingly.

The predicted background signal for each TE was estimated by subtracting the sum of all predicted metabolite signals, including water, from the original input data. This residual background was also scaled using a prior distribution. Notably, the background includes macromolecule signals, noise, and spectral artifacts. All prior distributions were modeled as normal distributions centered at the predicted values, with relative standard deviations set as a percentage of their respective means. The scaled component signals were subsequently summed to synthesize each test dataset. Datasets generated in this way were then reprocessed by deepJPRESS, yielding sensitivity intervals of the target concentration estimates.

Further resampling of variations in phase, frequency offset, and lineshape was omitted, since the model was trained to be invariant to these PFL factors. A total of 30 resampled test datasets were generated for each evaluation.

### 2.8 Training Setup

The deepJPRESS model was trained using Google TPU v2.8. Training data was generated in real‐time as a preprocessing routine before being fed to the model. The training loop consisted of 18 epochs and took approximately 22 h to complete. The model weight parameters were updated using an Adam optimizer and a cosine annealing scheduler with a maximum learning rate of 5 × 10^−4^. All loss functions utilized mean absolute errors. Negative predicted concentrations and FID amplitudes were clipped to zero before computing the loss functions. This clipping, which is mathematically equivalent to ReLU, was implemented inside the loss function, allowing it to remain an optional post‐processing step during test time.

## 3 Results

**FIGURE 3:**
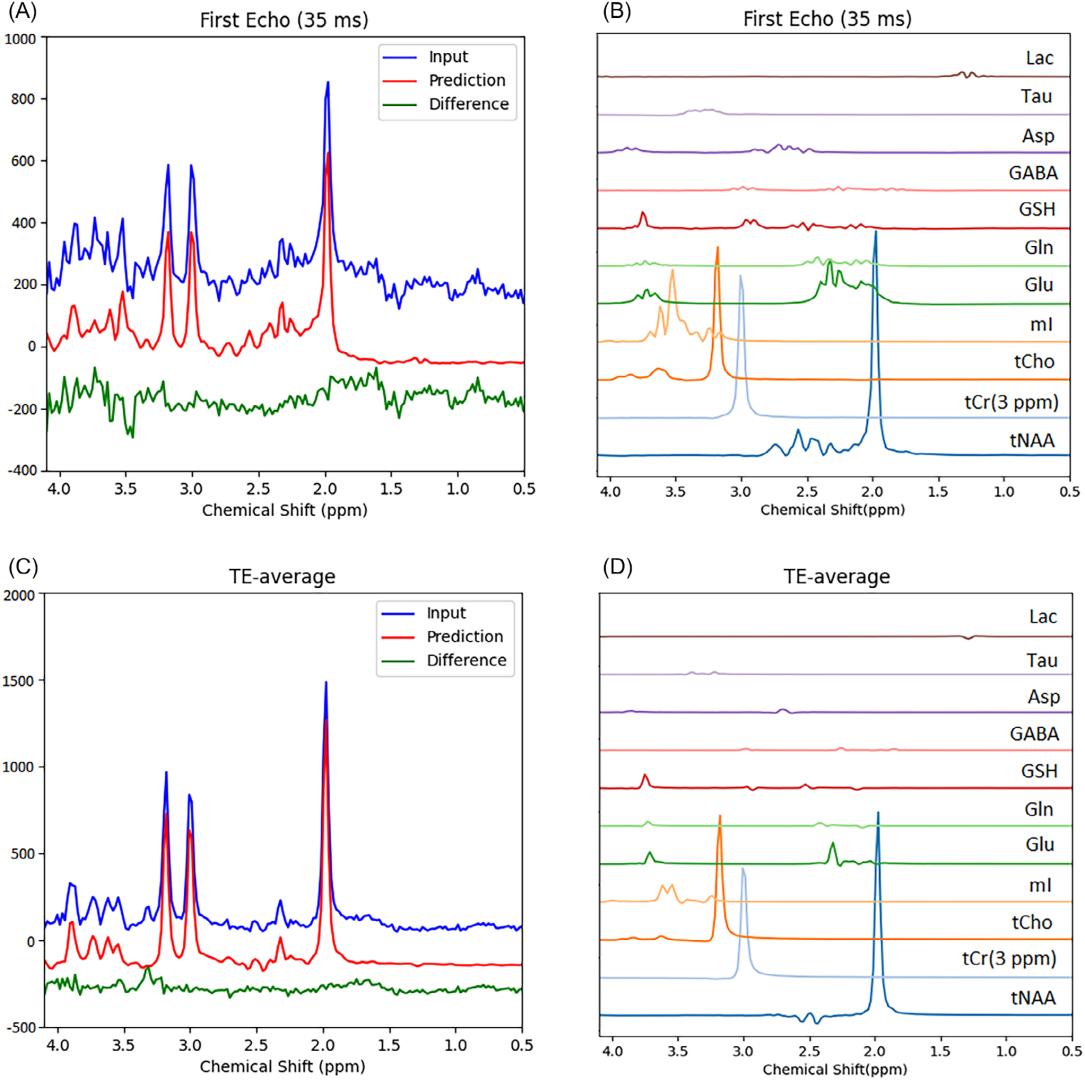
Representative spectral predictions using in vivo JPRESS data from a single participant. Left column: First‐echo spectrum (35 ms) and echo‐time–averaged spectrum. Blue: In vivo input spectra; Red: Predicted spectra; Green: Difference spectra. Right column: Predicted individual component spectra for the first echo and the average over 32 echoes.

The predicted first‐echo spectrum and the spectrum averaged across all 32 echoes (TE‐averaged spectrum) [14, 16] were compared with their respective input spectra, as shown in the left column of Figure 3. The predicted spectra of individual components are displayed in the right column. The predicted spectra in the left column were obtained by summing all predicted individual component FIDs, including the residual water signal, followed by Fourier transform. The difference lines represent the “residuals,” or the differences between the predicted and input spectra. Despite the presence of strong spike artifacts in the down‐field region of the first echo spectrum, presumably caused by outer‐volume signals, which disappeared in our acquisitions after the crusher gradients for outer volume suppression had been rearranged, the model successfully isolates the target metabolite signals. In the TE‐averaged spectrum, the residuals show unregistered peaks between 3 and 3.5 ppm. The small “blips” near 3.3 and 3.6 ppm were assigned to scyllo‐inositol and glycine, respectively. Both scyllo‐inositol and glycine were not included in the training and therefore were filtered out by the model. These residual peaks have also been observed in other datasets (see Figure S1). The predicted metabolite concentrations and T_2_ values for the 30 in vivo datasets were listed in Table 1. For reference, the corresponding concentration ranges reported in the literature were also included in the table.

**TABLE 1:** Metabolite concentrations (mM) and transverse relaxation T_2_s (ms) predicted by deepJPRESS with 30 JPRESS datasets acquired from the anterior cingulate cortex of healthy participants.

	tNAA	tCr	tCho	mI	Glu	Gln	GSH	GABA	Asp	Tau	Lac
Mean Conc.	9.99	7.50	1.75	7.10	10.0	2.04	1.56	0.68	2.85	1.27	0.66
Reference	8.4–12.3	6.5–8.0	1.5–2.2	5.5–7.5	8.0–10.7	1.6–2.5	1.2–2.3	0.3–1.1	2.9–3.8	1.0–1.5	0.6–1.0
Conc. Std.	0.56	0.52	0.18	0.92	0.78	0.36	0.41	0.22	0.58	0.50	0.24
Mean T_2_	247	204	234	266	192	176	212	227	195	254	242
T_2_ Std.	14	10	13	17	8	8	12	15	10	16	13

Abbreviations: Asp, aspartate; GABA, gamma‐aminobutyric acid; Gln, glutamine; Glu, glutamate; GSH, glutathione; Lac, lactate; mI, myo‐inositol; Tau, taurine; tCho, total choline and phosphocholine; tCr, total creatine and phosphocreatine; tNAA, total N‐acetylaspartate and N‐acetylaspartylglutamate.

**FIGURE 4:**
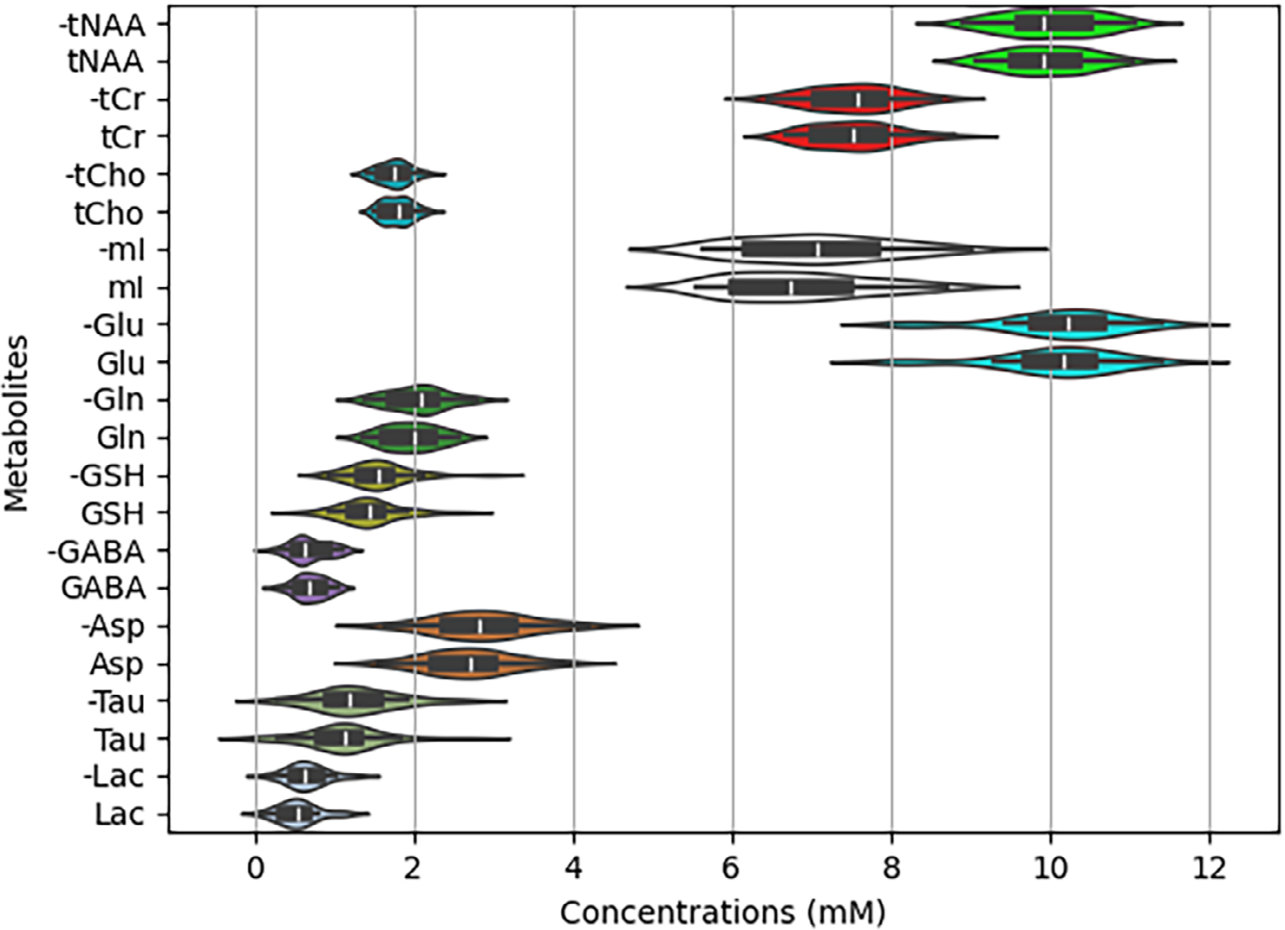
Comparisons of the distributions of the predicted metabolite concentrations across the 30 in vivo datasets before (marked by “‐” in front of the metabolite names along the vertical axis) and after the datasets were severely disturbed by random phase shifts, frequency offsets, and spectral linewidth broadening. The phase shifts (from −180° to 180°) and frequency offsets (from −5 Hz to 5 Hz) were applied independently across 32 free induction decay (FID) signals, while all FIDs within a dataset shared the same Voigt‐type broadening with the Lorentzian parameter ranging from 0 to 5π s^−1^ and the Gaussian parameter from 0 to 15.5 s^−2^. The box‐violin plots display both data distribution and summary statistics.

Figure 4 compares the distributions of predicted metabolite concentrations before and after applying PFL perturbations, with the perturbed cases shown directly below the corresponding unperturbed cases. As described in the Methods section, the perturbations included phase shifts ranging from −180° to 180°, frequency offsets from −5 to 5 Hz, Lorentzian broadening from 0 to 5π s^−1^, and Gaussian broadening from 0 to 15.5 s^−2^. Figure 4 demonstrates that these large PFL perturbations resulted in only minimal effects on the distributions of predicted concentrations over the 30 in vivo datasets.

**FIGURE 5:**
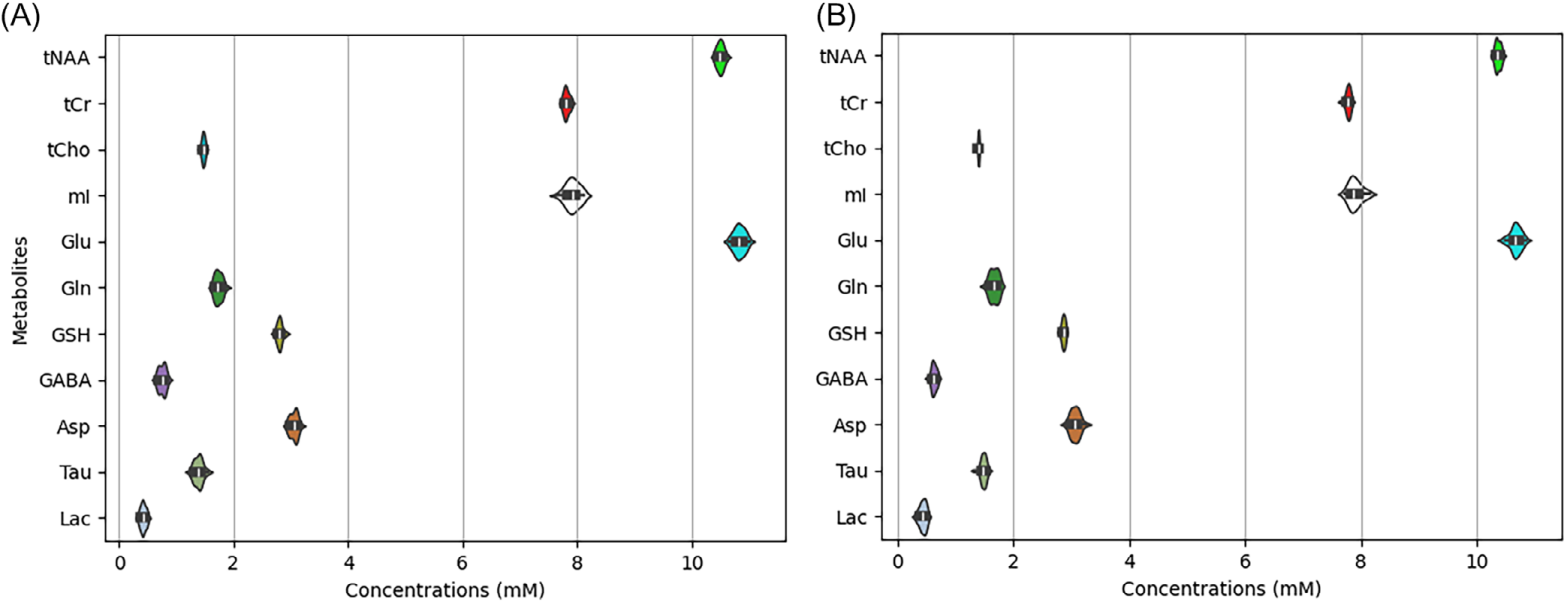
Influence of phase (left) and frequency offset (right) on the predicted concentrations. Random phase shifts and frequency offsets were applied to the 32 TE‐specific free induction decay (FID) signals of an individual in vivo dataset, resulting in 30 datasets with phase shifts ranging from −180° to 180° and another 30 datasets with frequency offsets ranging from −5 Hz to 5 Hz.

Predicted metabolite concentrations were further evaluated after applying random phase and frequency shifts to the 32 TE‐specific FIDs of an individual in vivo dataset, resulting in 30 datasets with phase shifts ranging from −180° to 180° and 30 datasets with frequency shifts from −5 to 5 Hz. As shown in Figure 5, only minor deviations were observed for both cases compared to the distributions in Figure 4. Additional examples are provided in Figure S2.

**FIGURE 6:**
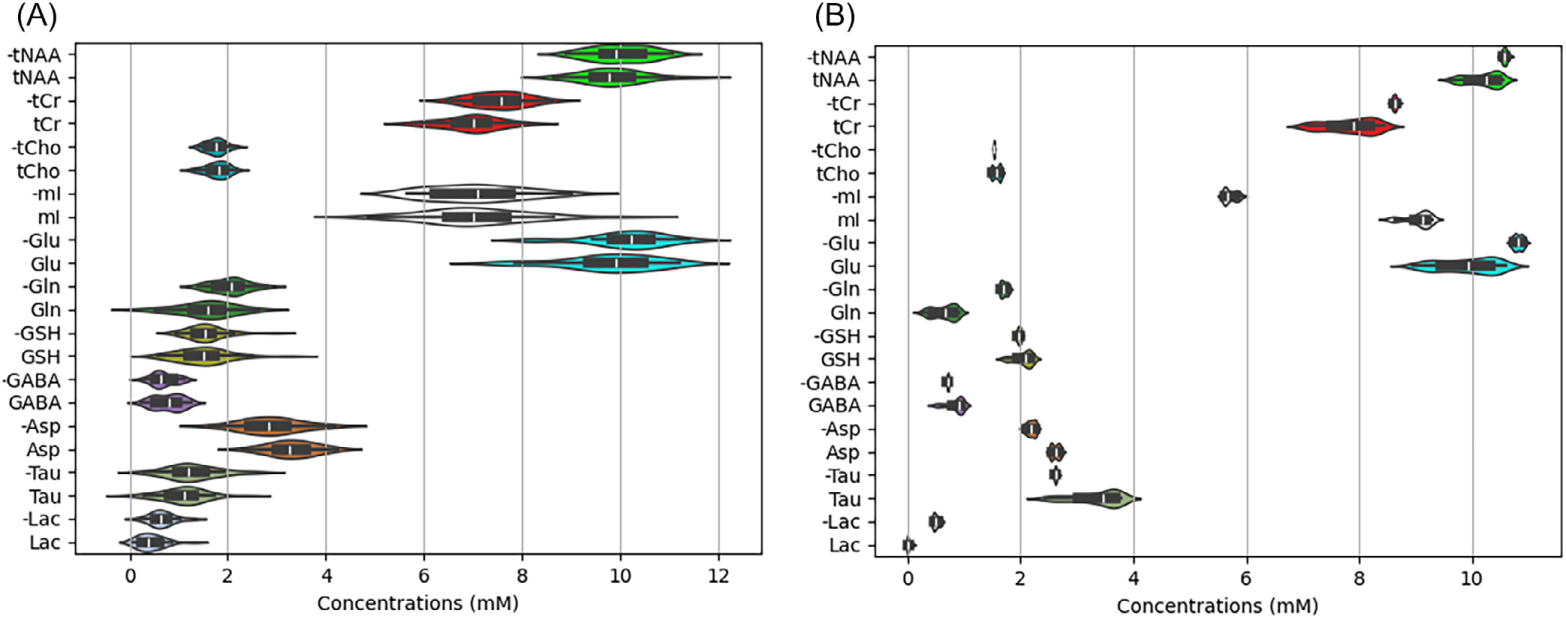
Comparison between pooling at the beginning (PAB) of the time‐domain signals and global pooling over all 2048 sampling points. PAB and global pooling results are shown with the former marked by the “‐” in front of the metabolite names on the vertical axis. Predicted concentration distributions are shown for the 30 in vivo datasets (left) and for 30 datasets generated by applying a Voigt‐type linewidth broadening function to a selected in vivo dataset, with random Lorentzian (from 0 to 5π s^−1^) and Gaussian (from 0 to 15.5 s^−2^) parameters. Overall, PAB reduced the spread of the distributions in both cases, with more pronounced improvements observed in the line‐broadened dataset derived from the single in vivo dataset, where inter‐subject discrepancies were avoided.

Lineshape invariance was achieved through the use of PAB. Comparisons between PAB and global average pooling (over all 2048 sampling points) are shown in Figure 6. The left panel shows predicted concentrations from the 30 in vivo datasets, while the right presents results from 30 datasets derived by applying a Voigt‐type linewidth broadening function to an individual in vivo dataset, where Lorentzian and Gaussian parameters were randomly drawn from very large ranges of 0 to 5π s^−1^ and 0 to 15.5 s^−2^, respectively. While PAB reduced the spread of the predicted concentration distributions in both cases, more pronounced improvements were observed for the 30 datasets derived from the seed dataset, where inter‐subject variability, such as water referencing and tissue segmentation differences, was non‐existent. Additional examples are provided in Figure S3. The modest deviations observed after the use of PAB demonstrate the robustness of the model over large lineshape variations.

**FIGURE 7:**
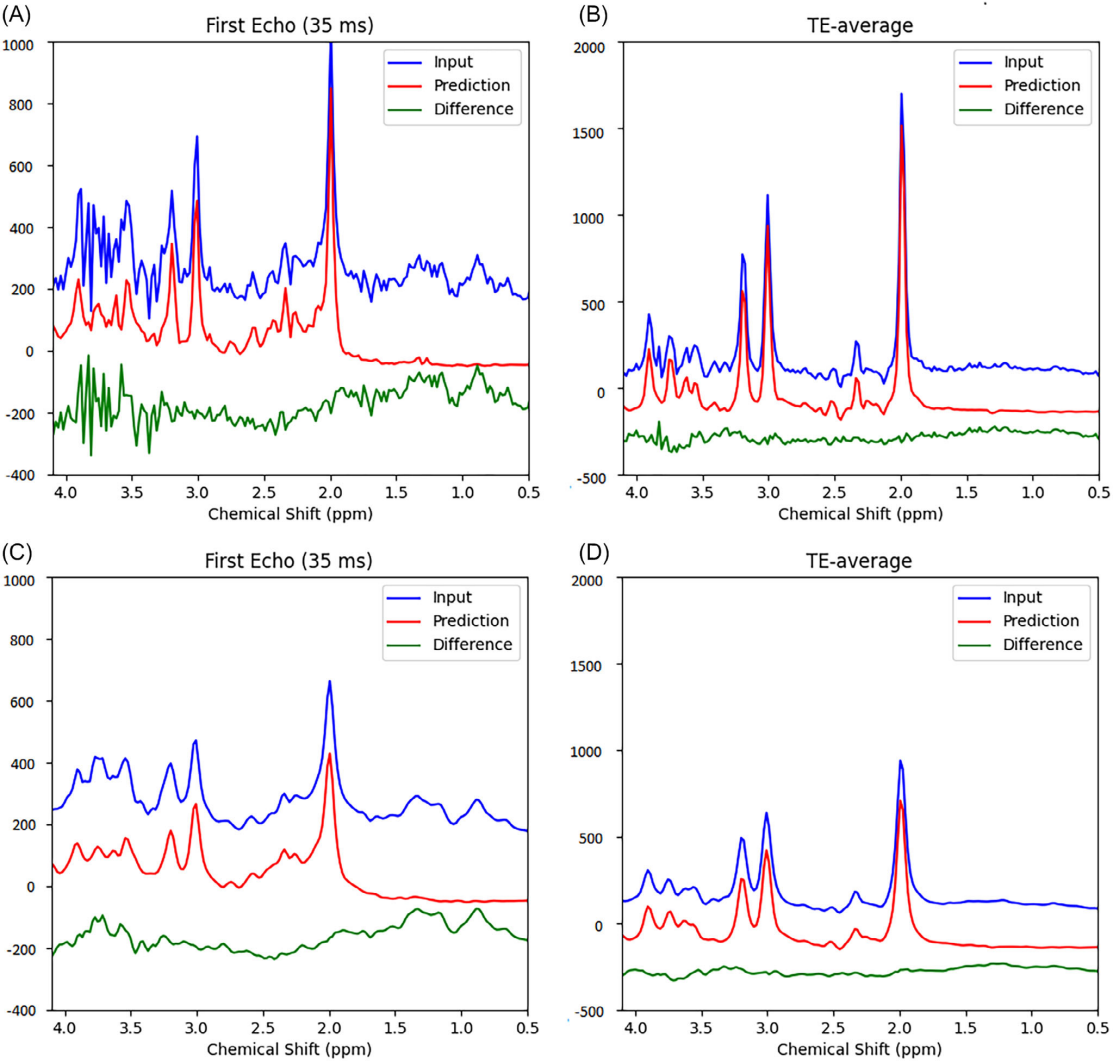
Comparison of predicted first‐echo and TE‐averaged spectra before (top panel) and after (bottom panel) applying lineshape distortions to the original input JPRESS data. The following parameters were used to generate asymmetric lineshape distortion in the bottom panel: 5π (Lorentzian term), 15.5 (Gaussian term), −1.55 (*b*_1_), 15.5 (*b*_2_), and 15.5 (*b*_3_).

Figure 7 demonstrates an example of severe asymmetric lineshape distortion, where the lineshape was altered using Equation (1) with the following parameters: 5π (Lorentzian term), 15.5 (Gaussian term), −1.55 (*b*_1_), 15.5 (*b*_2_), and 15.5 (*b*_3_). The predicted concentrations before and after the lineshape change are: tNAA [10.4 (before), 10.4 (after)], tCr [7.83, 7.85], tCho [1.40, 1.43], mI [8.20, 8.05], Glu [10.64, 10.67], Gln [1.65, 1.42], GSH [2.96, 2.73], GABA [0.64, 0.73], Asp [3.20, 2.82], Tau [1.50, 1.35], and Lac [0.45, 0.35]. These changes are consistent with the distributions shown in Figure 6.

**FIGURE 8:**
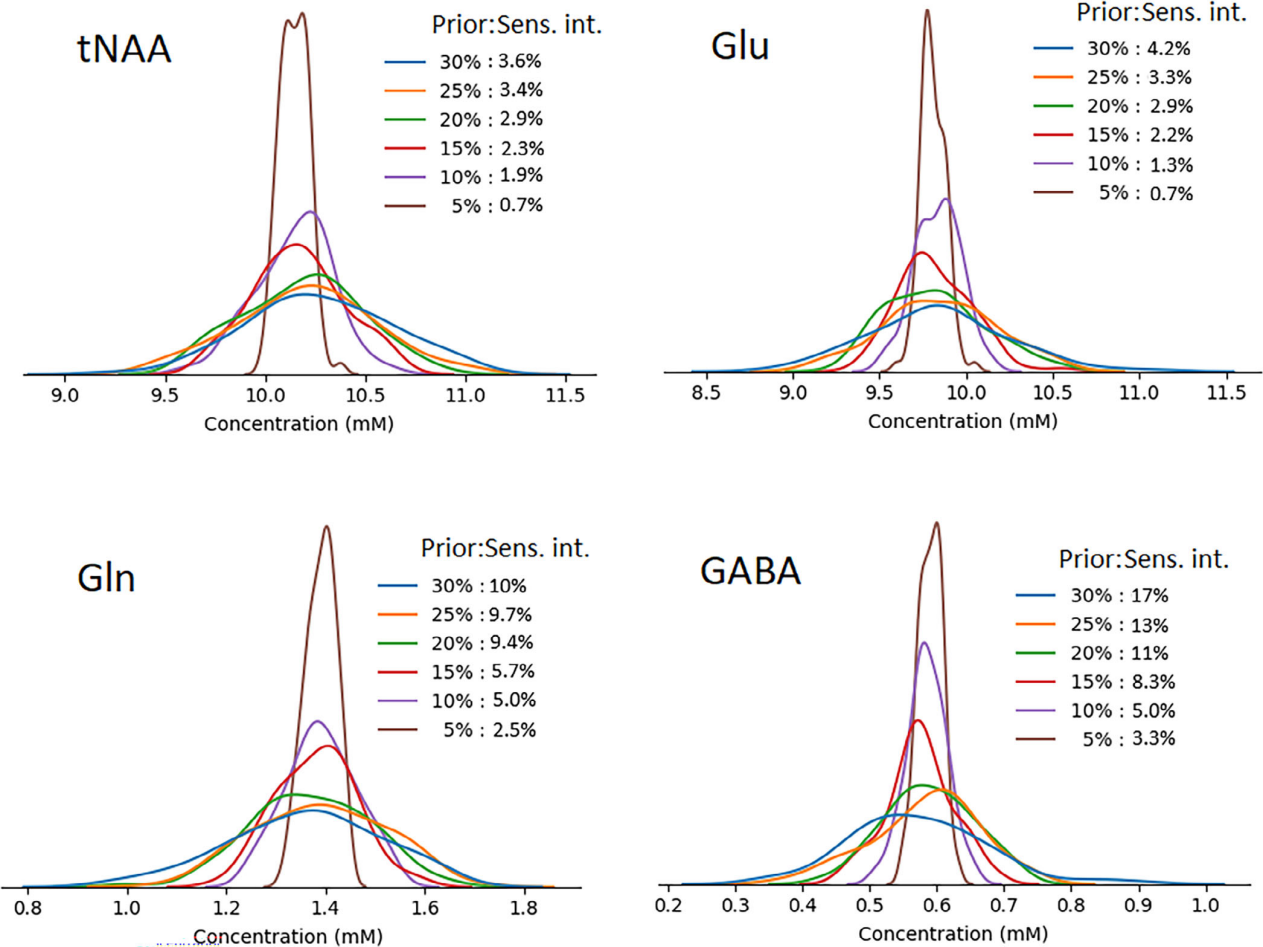
Sensitivity intervals (Sens. int.) of the predicted concentrations of tNAA, Glu, Gln, and GABA are shown for five prior distributions applied to resampled test datasets, with standard deviations ranging from 5% to 30% relative to the predicted concentrations of the remaining components, including residual water and background signals. The resulting sensitivity intervals are substantially smaller than the corresponding prior uncertainties, demonstrating stable and robust model performance.

To demonstrate the assessment of prediction uncertainty through data resampling, Figure 8 presents the sensitivity intervals of tNAA, Glu, Gln, and GABA concentrations resulting from prior uncertainties in the remaining model components. Five prior distributions were tested, with standard deviations ranging from 5% to 30% relative to the predicted concentrations of individual components, including residual water and background signals. As expected, greater uncertainty in the remaining components led to increased variability in the predicted target concentrations. However, the sensitivity intervals of the target predictions were substantially smaller, in relative terms, than those of the remaining components, indicating stable and robust model performance. Four examples of resampled spectra and the corresponding predicted FIDs are provided in Figure S4, showing that the Glu signal remained consistent with the original prediction, whereas other components and background signals were randomly scaled using a normal distribution with a relative standard deviation of 25%.

**FIGURE 9:**
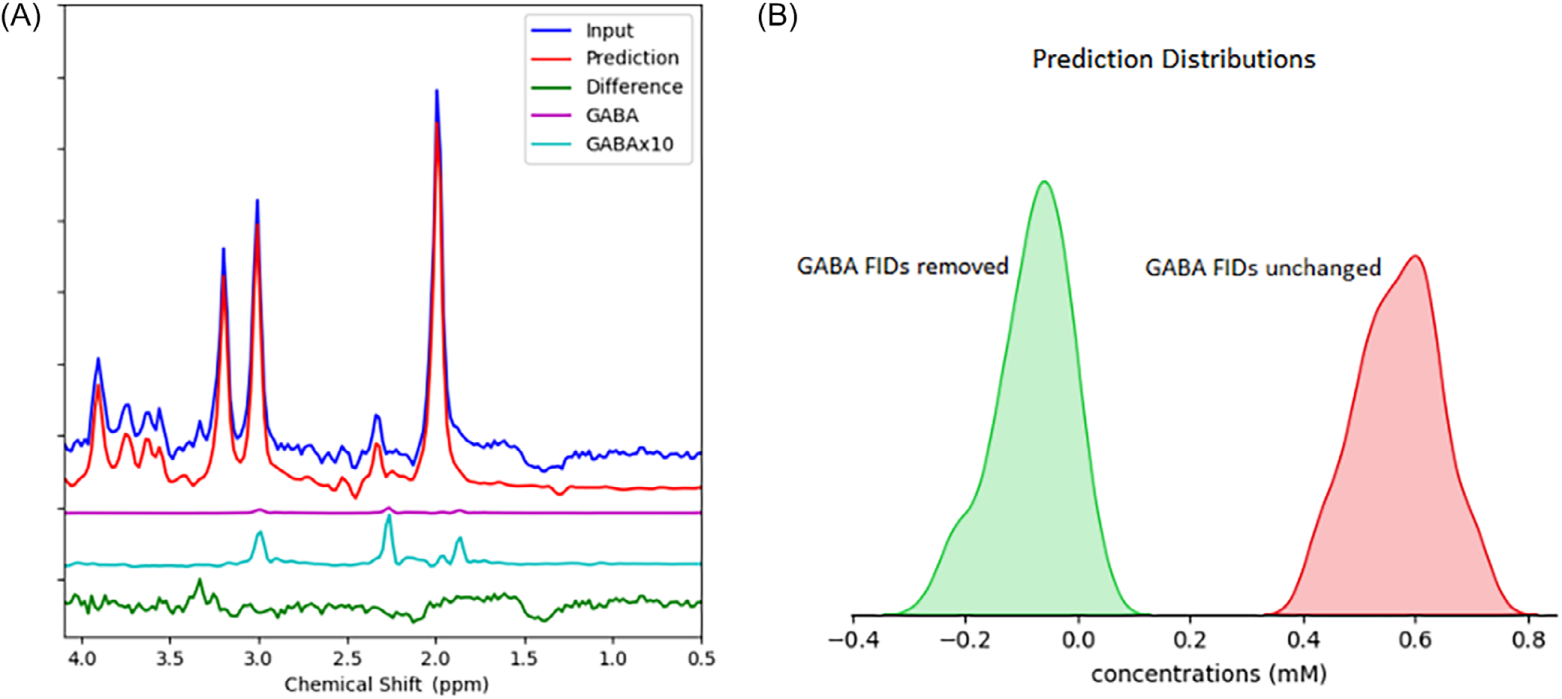
(a) TE‐averaged spectra with the GABA component highlighted. Blue: In vivo TE‐averaged spectrum from an individual participant; Red: Predicted TE‐averaged spectrum; Purple: Predicted GABA component at scale; Cyan: Predicted GABA spectrum ×10. The green line represents the residuals obtained by subtracting the predicted spectra (red) from the input spectra (blue). (b) Distributions of the predicted GABA concentrations for two regenerated datasets of 100 samples each: Without (green) and with (red) the predicted GABA FIDs.

To illustrate the model's capability in detecting GABA, a predicted TE‐averaged spectrum along with the in vivo input and the predicted spectra of all target metabolites are shown in Figure 9A. The predicted GABA concentration is 0.56 mM. In Figure 9A, the predicted GABA signal is further highlighted by 10‐fold upscaling. Figure 9B illustrates the distributions of predicted GABA concentrations for two cases using resampling‐based regenerated data: one where the originally predicted GABA FIDs were removed (left) and the other where they remained unchanged (right). For each case, 100 datasets were regenerated, with all other predicted component FIDs and background signals varied randomly. In the cases where the GABA FIDs were removed, the predicted GABA concentrations are expected to be 0. Figure 9B demonstrates that the deep learning model is sufficiently sensitive to GABA level and therefore can predict its concentration using JPRESS data without spectral editing.

## 4 Discussion

Deep learning has recently been increasingly applied to in vivo MRS. Most current studies of deep learning MRS focus on data preprocessing tasks such as denoising, spectral reconstruction, correcting phase shifts and frequency drifts, or removing backgrounds originating from macromolecules and spectral artifacts [17, 18, 19, 20, 21, 22, 23, 24]. There have also been approaches in which spectral fitting is aided or combined with deep learning to perform quantification [25, 26, 27, 28, 29, 30, 31, 32]. This study demonstrates that both metabolite concentrations and T_2_ can be directly predicted using the JPRESS representation through deep learning. From a neural network architecture perspective, the deepJPRESS model combines the strengths of manifold learning and representation learning, mapping the input into a latent space while reconstructing individual component FIDs. It takes a series of TE‐specific spectra in the time domain as input, with the encoder trained to capture spectral features such as chemical shifts and spin coupling patterns. The model learns to identify target signals and filter out unregistered signals, eliminating the need to model macromolecules and/or spectral artifacts including scalp lipids, and removing the requirement for auxiliary information (e.g., inversion‐recovery spectra) to be incorporated into JPRESS spectral quantification [33, 34].

Although the deepJPRESS model was trained on synthesized data, where metabolite concentrations and T_2_ values varied widely with uniform distributions and without prior knowledge of actual in vivo concentration distributions, the test results from the 30 in vivo datasets show that the predicted metabolite concentrations (Table 1) align well with values reported in the literature [35, 36, 37, 38, 39, 40, 41, 42, 43, 44, 45] and/or references cited therein. The metabolite concentration ranges in Table 1 were obtained from the literature after removing extreme values. The predicted T_2_ values also fall within the ranges reported in studies conducted at 3 Tesla using various MRS methods across different regions of the human brain [45, 46, 47, 48, 49, 50, 51, 52]. Additionally, we have also applied the deepJPRESS model to clinical studies involving voxels in different brain regions and hardware configurations.

PFL invariance is a critical property of the proposed deep learning model in this study that helps prevent overfitting during training and eliminates the prediction uncertainties caused by variations in spectral phase and frequency offsets. The results shown in Figures 5, 6, 7, Figures S2 and S3 confirm its effectiveness when applied to in vivo data, demonstrating that uncertainty induced by PFL was minimized. Our model training on synthesized datasets also revealed that a shared set of lineshape parameters was required across all 32 TEs as expressed in Equation (3). Achieving lineshape invariance requires the input data to retain sufficient spectral information to accommodate arbitrary lineshape parameters. If the linewidth becomes too broad, the early FID points may no longer capture enough spectral information, resulting in a weakened representation. Nevertheless, as shown in Figure 6 (right), although the prediction variations due to linewidth broadening were noticeable, these variations were greatly reduced when the model incorporated PAB. Given the large range of linewidth variations employed in Figures 6 and 7, our results demonstrate that deepJPRESS is well‐suited for application to in vivo data acquired in clinical settings.

The proposed PAB is a new approach to aggregating information with decaying signals. PAB offers two key benefits: first, the model only needs to determine the amplitudes of the FID signals at the initial points, which are directly linked to the concentrations of the targets, whereas global pooling involves all sampled points, which are heavily influenced by spectral lineshapes; secondly, PAB simplifies the process of determining T_2_ by minimizing the complicating factor of field inhomogeneity. This approach enhances the model performance, which was observed during training with synthesized data. Specifically, the concentration loss and FID reconstruction loss converged smoothly with the use of PAB, whereas global average pooling caused competition between minimizing these two losses, leading to less effective convergence. A comparison of the concentration and FID losses during model training with PAB and global average pooling is provided in Figure S5.

Rescaling the outputs of the JPRESS representation using given prior distributions provides a unique resampling‐based method for assessing prediction uncertainties. Unlike model ensemble methods, which capture uncertainty arising from variability in model parameters or architectures [25], this novel resampling approach emphasizes data‐driven variability by perturbing the input space while keeping the model fixed. As shown in Figure 8, the sensitivity intervals for the target concentration are substantially smaller than the corresponding prior distributions for the remaining components. This indicates that deepJPRESS has a high degree of confidence in its predictions, and that the prior distribution can serve as a conservative upper bound when assessing the uncertainty of the target concentration. For example, setting the standard deviation of the prior distribution to 25% of the predicted concentration defines a relatively broad prior belief. In Figure 8, the predicted Glu concentration is 9.8 mM, with a relative standard deviation of 4%, corresponding to an absolute error of 0.4 mM. This variability, substantially lower than the variability observed across the 30 in vivo datasets summarized in Table 1, reflects the robustness of deepJPRESS to input perturbations. This reduced prediction variance suggests that much of the uncertainty reported in Table 1 likely stems from inter‐subject differences, water reference scaling (Equation 5 in Ref. [6]), and tissue composition heterogeneity, rather than from any intrinsic prediction error.

A neural network model can exhibit much greater sensitivity than human vision due to its ability to learn deep correlations across the data. The GABA signal is notably weak because of its low concentration, as shown in the predicted TE‐averaged spectrum in Figure 9A. It is important to note that metabolite concentrations were determined using spectra acquired at 32 different TEs and that the TE‐averaged spectra were used in Figure 9A only for visualization purposes. While the presence of GABA may be difficult to discern visually in the JPRESS spectral data due to overlapping and stronger signals, deepJPRESS reveals a clearly measurable difference caused by the removal of the GABA signal, as illustrated in Figure 9B.

Deriving transverse relaxation time for a spin‐coupling system is a complex process, particularly when a molecule contains different moieties or subgroups with different T_2_ values, which cannot be fully resolved through simple model fitting. Artificial neural networks offer the advantage of encoding complex relationships with a large set of learned parameters. However, challenges arise in creating accurate T_2_ labels when multiple T_2_ values for a single metabolite are involved. We addressed this difficulty by training the model to learn the T_2_‐averaged across all subgroups of a target metabolite, with results listed in Table 1 in agreement with literature reports [45, 46, 47, 48, 49, 50, 51, 52].

The extraction of metabolite T_2_ could be improved in future studies by training the model to directly learn the individual T_2_ values of all subgroups within a metabolite with in vivo data acquired at higher magnetic field strengths. For this purpose, the metabolite FIDs would need to be decomposed into subgroups, ensuring that the signal in Equation (2) only contains a single T_2_ component with an independent $\delta_{2}$term when applying Equation (3). Another advantage of FID decomposition is that it allows for independent variations in chemical shifts of subgroups within a metabolite to account for the heterogenous susceptibility landscape of the in vivo environment. Since FID decomposition is expected to better account for differences between simulated data and in vivo spectra including differences in their subgroups, future studies will explore these advantages.

Figures S6 and S7 compare the widely used in vivo spectral fitting software LCModel with deepJPRESS. In Figure S6, 10 datasets were generated by resampling a selected in vivo JPRESS dataset. After processing the data with deepJPRESS, the predicted individual component FIDs were randomly scaled within a range of 50% to 150% of their predicted values and then summed to generate the input datasets for comparison with LCModel. The scaled values, relative to tCr, were plotted on the horizontal axis, while the concentrations estimated by LCModel and deepJPRESS were plotted on the vertical axis. Despite producing visually good spectral fits, LCModel exhibited strong systematic errors with respect to the true concentration variations at the very first TE. In Figure S7, the same original in vivo dataset was tested under varying spectral linewidths, broadened from 0 to 4.5 Hz. The tNAA concentration estimated by LCModel decreased significantly with increasing spectral linewidth (Figure S7a). A less pronounced dependence was observed for the tNAA/tCr ratio (Figure S7b), suggesting that the tNAA/tCr ratio partially mitigated the effects of lineshape mischaracterization by LCModel. This artificial correlation between concentration estimates and spectral linewidth generated by LCModel was also noted for other metabolites. As shown in Figure S7c,d, the LCModel‐generated baseline exhibited strong undulations as spectral linewidth increased. These large artificial baseline variations for the same in vivo data are clearly non‐physical, contributing to substantial spectral fitting errors. In contrast, deepJPRESS demonstrated high robustness to linewidth variations, producing stable concentration estimates and consistent reconstructed FID signals. The predicted background also remained stable across all conditions, highlighting the resilience of deepJPRESS against spectral distortions that often compromise LCModel. Furthermore, ProFit and FitAID [33, 34] have been used to fit JPRESS data using experimentally determined 2D spectral baseline. In contrast, the deepJPRESS model does not require a priori knowledge of the spectral background as the background signals including the complex J coupling evolution of macromolecules over varying TEs are filtered out by our approach.

As a limitation of this study, we could not compare deepJPRESS with FitAID [33] or ProFit [34] because, while these spectral fitting methods also exploit multi‐TE information in JPRESS, they require additional inversion‐recovery spectra at each TE.

Finally, a substantial advantage of deepJPRESS is that it was trained without relying on prototypical in vivo spectral information, in contrast to conventional spectral fitting‐based quantification methods. Spectral fitting involves a non‐linear optimization process that requires the initialization of multiple fitting parameters. Achieving an optimal solution depends heavily on how close the initial settings are to the true values. Typically, spectral fitting methods use default initializations based on representative in vivo metabolite concentrations and lineshapes. However, large quantification errors can arise when these defaults do not match the characteristics of the actual data. deepJPRESS eliminates the data preprocessing process required by spectral fitting to set up the initial phase and frequency offset, allowing these parameters to vary across different TEs.

## 5 Conclusions

The JPRESS representation provides a high‐level depiction of spectral structures, directly mapping to both metabolite concentrations and T_2_ values. The in vivo results presented in this study demonstrate that the deepJPRESS model yields reliable predictions for both concentrations and T_2_s. Importantly, this technique does not rely on prior knowledge of unknown or TE‐dependent background signals, including spectral artifacts and macromolecular contributions. As such, it offers a robust and practical approach for determining neurochemical concentrations and T_2_s in vivo.

## Funding

This work was supported by the Intramural Research Program at the National Institute of Mental Health, National Institutes of Health (IRP‐NIMH‐NIH) (ZIA MH002803). The contributions of the NIH author(s) were made as part of their official duties as NIH federal employees, are in compliance with agency policy requirements, and are considered Works of the United States Government. However, the findings and conclusions presented in this paper are those of the author(s) and do not necessarily reflect the views of the NIH or the U.S. Department of Health and Human Services.

## Conflicts of Interest

The authors declare no conflicts of interest.

## Supporting information


**Data S1:** Supporting Information.

## Data Availability

The source code of deepJPRESS model is available at: https://github.com/MRS‐NIH/DeepJPRESS. The model parameters and in vivo data can be downloaded from: https://www.nitrc.org/frs/download.php/17158/fingerprint_model.h5 and https://www.nitrc.org/frs/download.php/18526/numpy_data.npz.
