# Supplementary material for: Spectral Representation of Neurochemicals With Phase, Frequency Offset, and Lineshape Invariance: Application to JPRESS for In Vivo Concentration and T2 Mapping by Deep Learning
Source: Magn Reson Med. 2026 Feb 7;95(6):3052–64. doi: 10.1002/mrm.70291 (PMC13049256; doi:10.1002/mrm.70291)

**Script S1**. TensorFlow (version 2.15) implementation of the proposed encoder and decoder*.

*See Ref. 6 for the implementation of WaveNet. Convolution kernel size = 5; dilation depth = 8; PAB points = 64; GRU hidden size = 128. Training loss used the following equation:

$$Loss=L_{conc}+L_{FID}+{L_{T2}+L}_{FID\_amp}$$

The four terms on the right-hand side were assigned weighting factors of 0.3, 20, 1, and 0.1, respectively.

**Table S1.** Data acquisition and processing.

| **1.Hardware**  a. Field strength: 3 T  b. Manufacturer: GE  c. Model: Signa  d. Proton single channel head coil  **2.Acquisition**  a. Pulse sequence: JPRESS  b. VOI location: anterior cingulate cortex  c. VOI size: 2x2x4.5 cm^3^  d. Repetition time: 3 s; echo time: 35 ms (first echo); number of echoes: 32; echo  spacing: 6 ms  e. Number of excitations: 4  f. Spectral width: 5000 Hz; sampling points: 4096  g. Water suppression method: CHESS  h. Shimming method: automatic first order shimming  **3.Data analysis**  a. Analysis software: in-house developed deep learning method  b. Only the first half of sampling points (2048) were used  c. Outputs: absolute concentration and T_2_ relaxation time  d. Gray and white matter water concentration: 43300 and 35580 mM; CSF T_2_: 2 s  **4.Data quality**  a. Typical SNR: 50 (NAA peak at 2 ppm after averaging); Typical linewidth: 7 Hz  (water)  b. Data exclusion criteria: None  c. CRLB, goodness of fit, and SD of residue: N/A  d. Sample spectra: see Figure S1 |
| --- |

**Figure S1.** Four additional examples of comparison between predicted and input spectra. For simplicity, only the first echo and echo-time averaged spectra are displayed. The predicted spectra were obtained by summing all predicted individual component FIDs, including the residual water signal, followed by Fourier transform. The difference spectra resulted from subtracting the prediction from the in vivo input. Blue: in vivo input; Red: prediction; Green: difference. Note the high consistency of the predicted spectra in spite of the large artefacts/variations across different in vivo spectra.


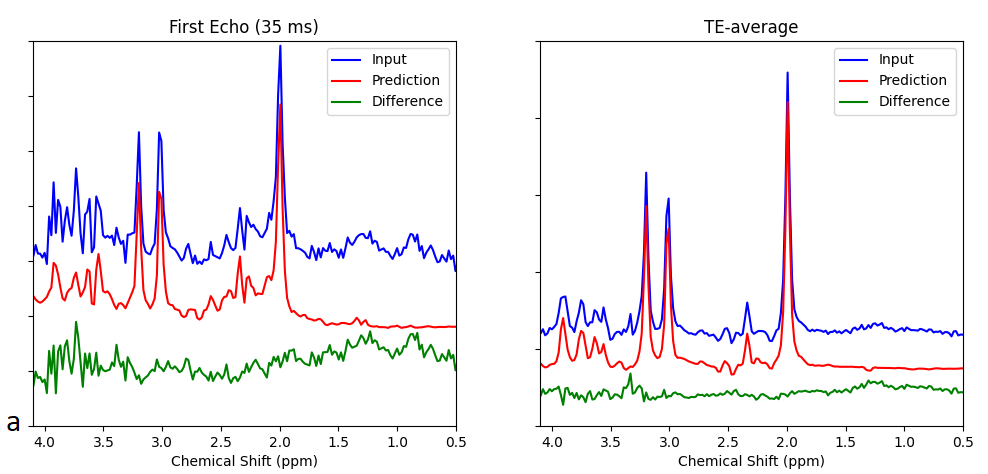


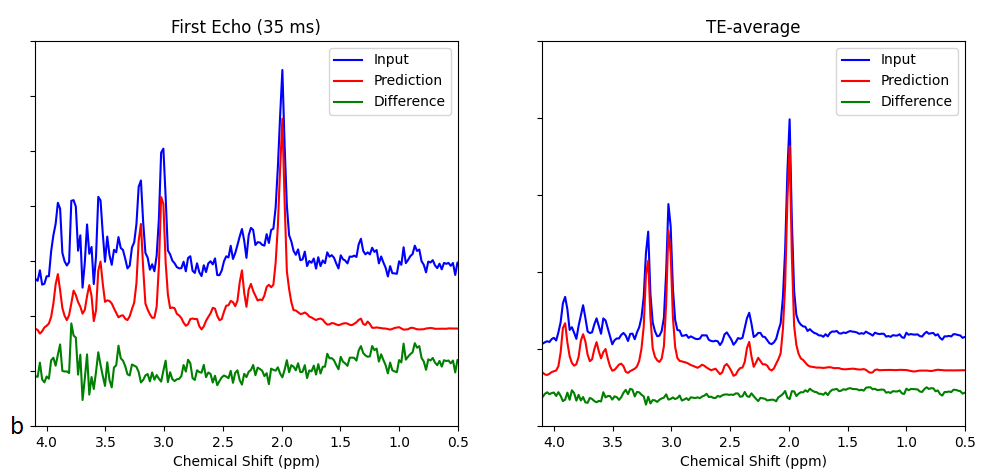


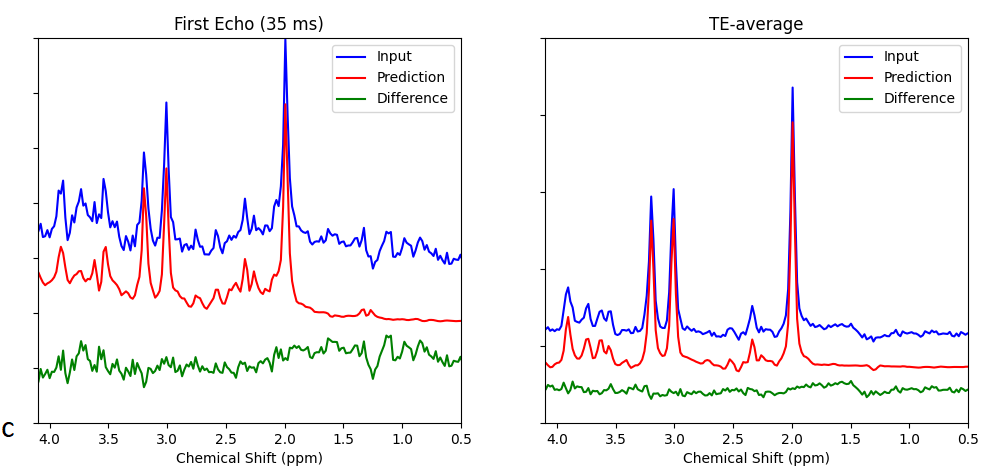


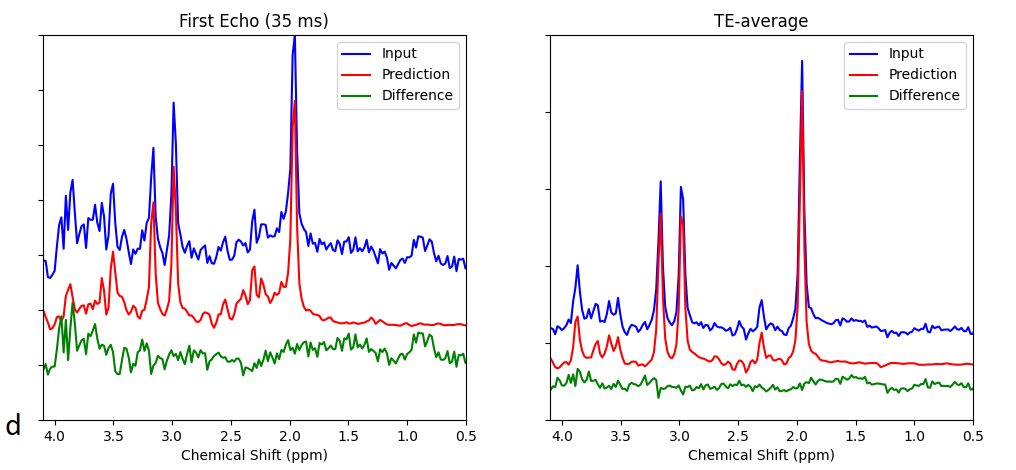


**Figure S2.** Four additional examples demonstrate that the effects of phase shifts and frequency offsets on predicted concentrations were minimized by training the model to be invariant to phase, frequency offset, and lineshape. Random phase shifts and frequency offsets, ranging from −π to π and −5 Hz to 5 Hz respectively, were applied independently to the 32 FIDs of each in vivo dataset.


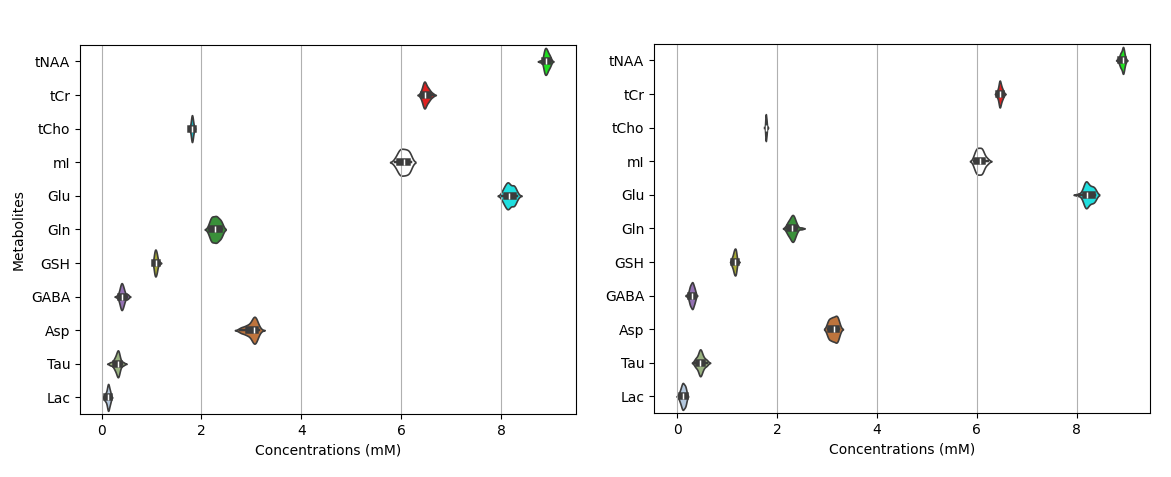


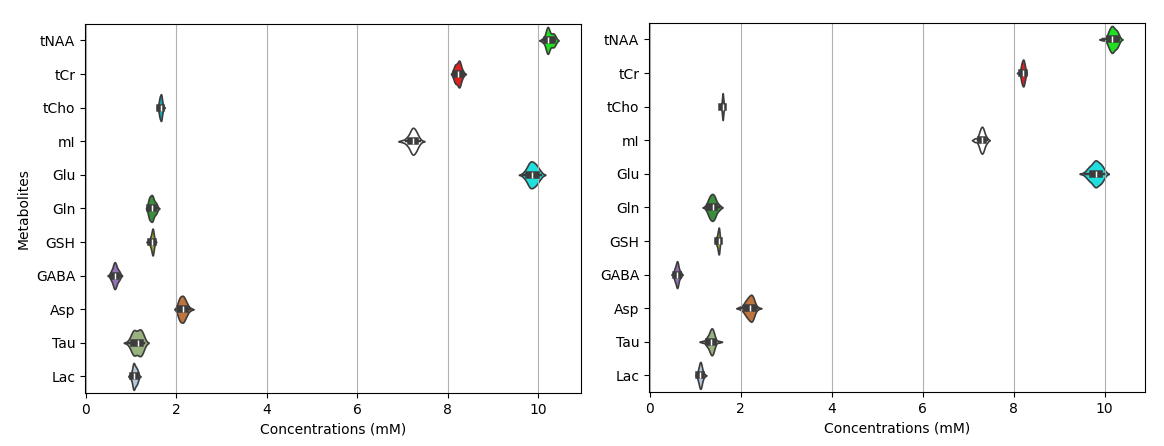


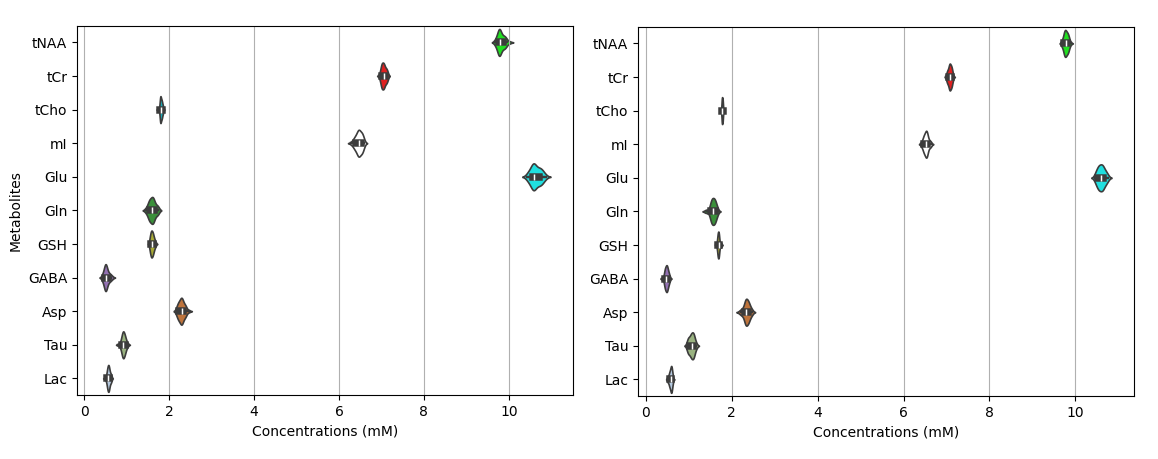


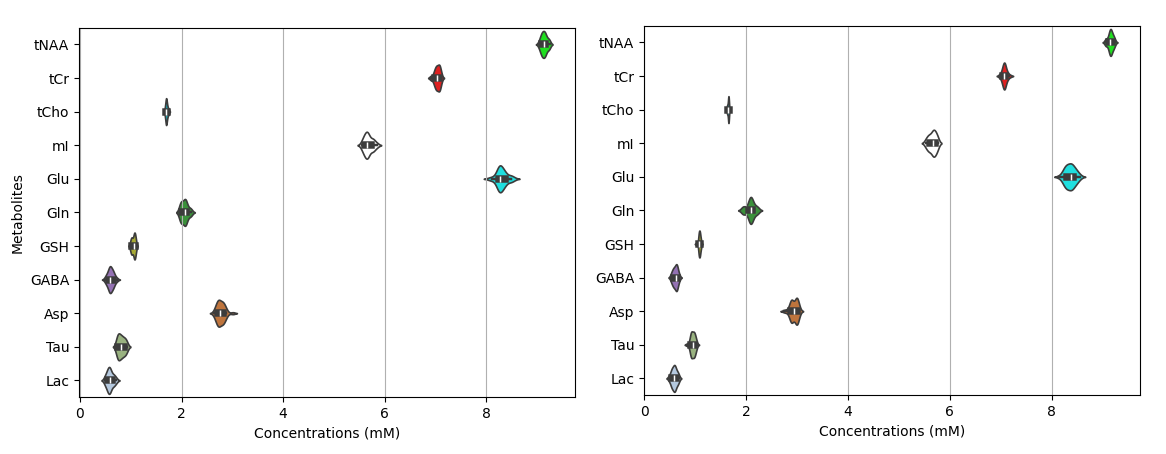


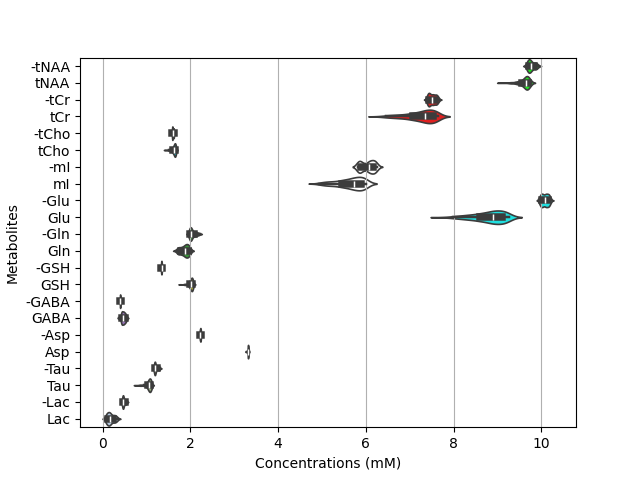

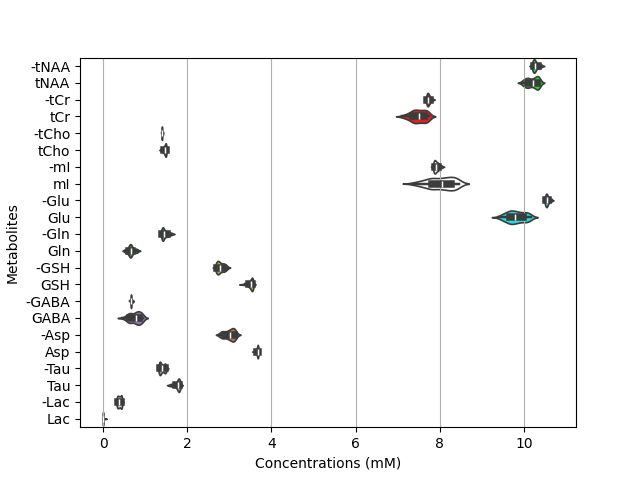
**Figure S3.** Four additional examples comparing PAB and global pooling using datasets generated from four individual in vivo JPRESS samples. The results for PAB and global pooling are presented with the former marked by “-” in front of the metabolite names along the vertical axis.


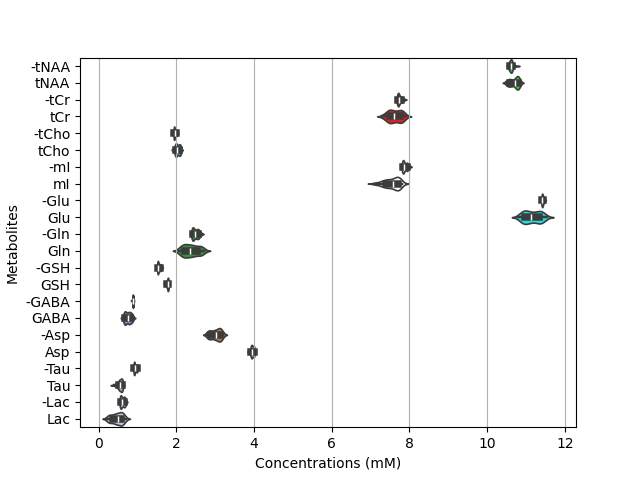

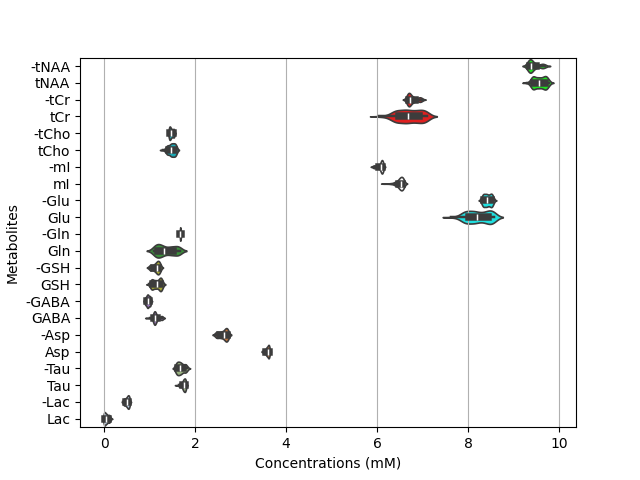


**Figure S4.** Four examples of resampled JPRESS spectra derived from an individual in vivo dataset. For simplicity, only the first TE and the TE-averaged spectra are displayed. During resampling, the glutamate signal remains consistent with the original prediction, while all other component FIDs, including the background, are randomly scaled according to a prior distribution. For the 100 regenerated samples, the mean and standard deviation of the predicted glutamate concentrations are 9.8 mM and 0.28 mM, respectively.

**
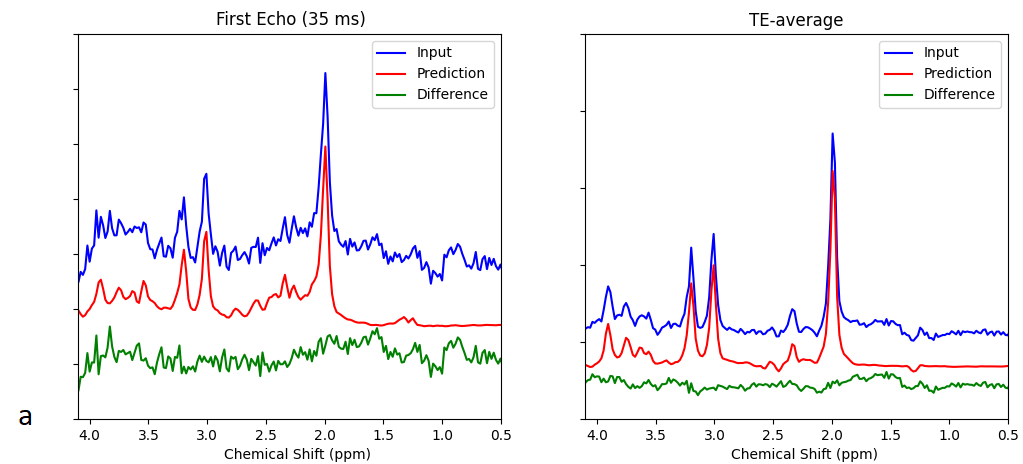
**

**
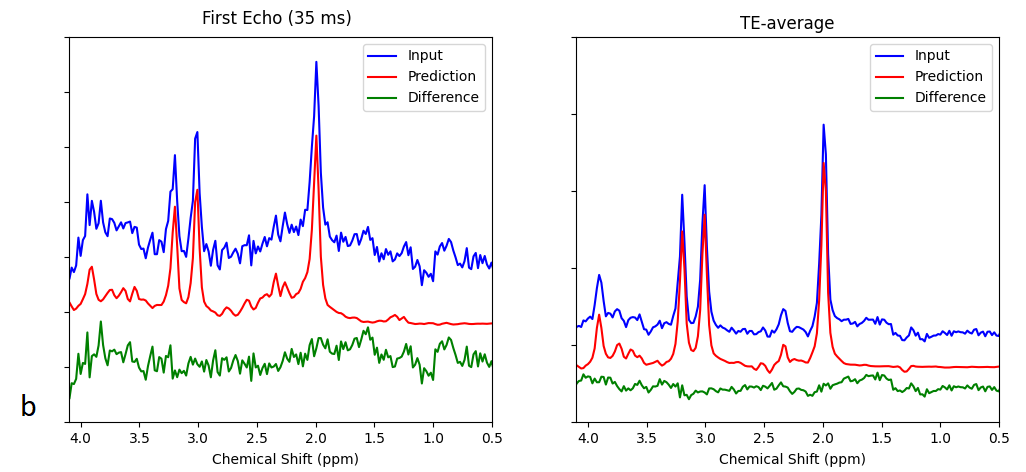
**

**
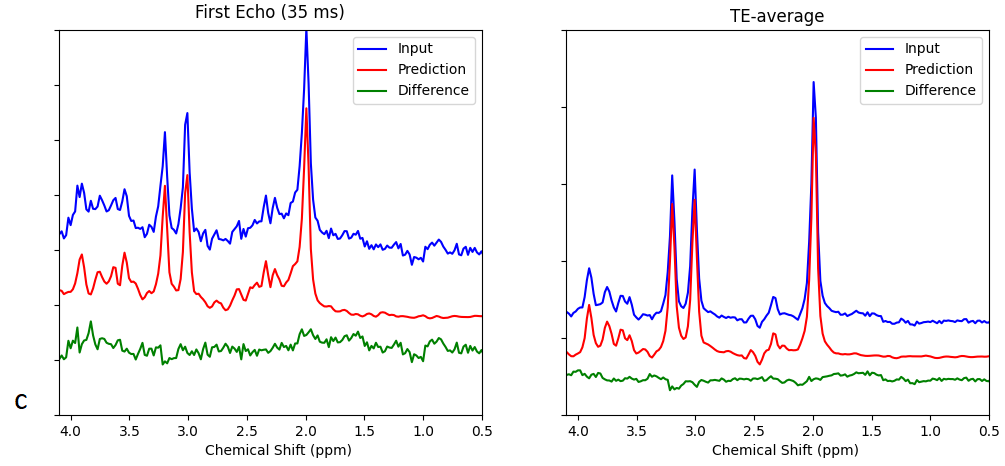
**

**
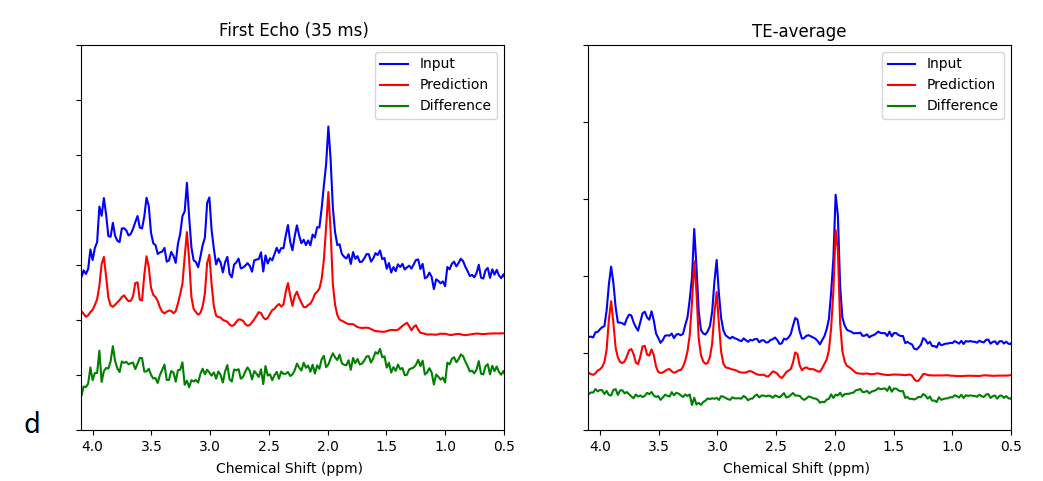
**

**Figure S5.** Comparison of training loss convergence with PAB and global average pooling. Left: Concentration loss. Right: Individual component FID loss. For PAB, three ablation experiments were performed with pooling points of 64, 128, and 256, respectively.


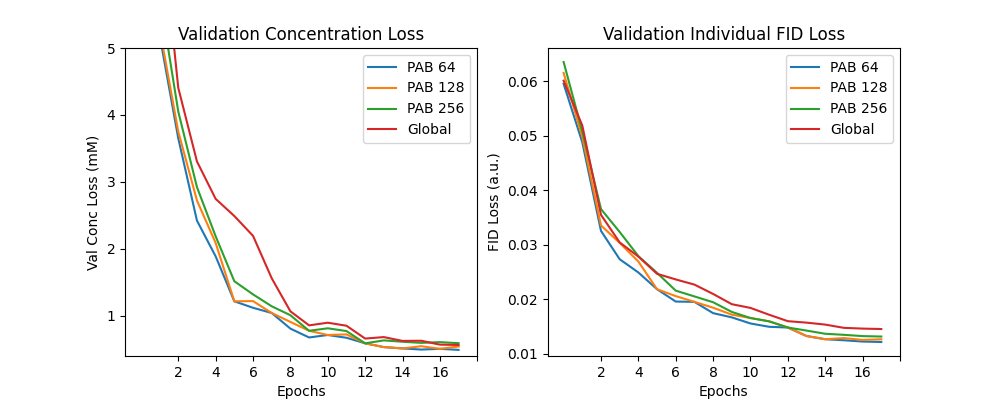


**Figure S6**: Ten datasets were generated by resampling an individual in vivo JPRESS dataset in order to avoid inter-subject variations in metabolite concentrations. Following the deepJPRESS processing, the predicted individual component FIDs were randomly scaled within a range of 50% to 150% of their predicted values and then summed to create ten datasets for comparison with LCModel. Data from the first TE were used as input to LCModel. The LCModel analysis was performed using default settings (version 6.3), including the vendor-provided macromolecule basis set. The resulting relative concentrations of tNAA, tCho, Glu, and Gln—expressed as ratios to tCr—are shown in panels a, b, c, and d, respectively, and are compared with the corresponding deepJPRESS predictions. The objective of this test was to assess the slope of the regression line between the estimated and true concentration ratio variations, where the correct value is 1. Four representative LCModel fits are displayed in panels e, f, g, and h, corresponding to datasets with tNAA/tCr ratios of 0.7, 1.0, 1.6, and 2.55, respectively. Despite visually good spectral fits, LCModel showed significant systematic errors when the relative amplitudes of individual component signals were varied as exemplified in panels a-d.


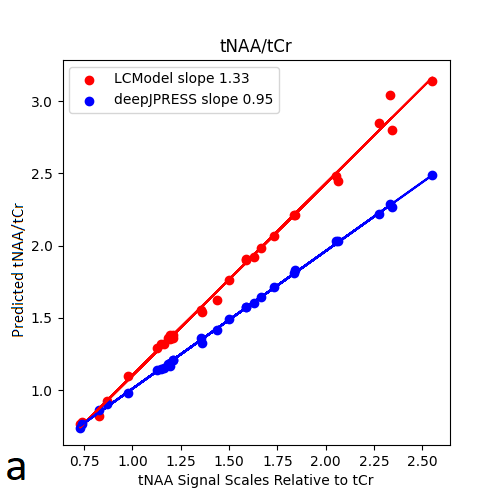

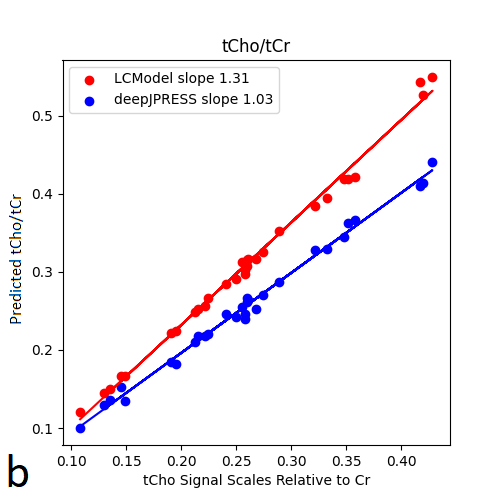


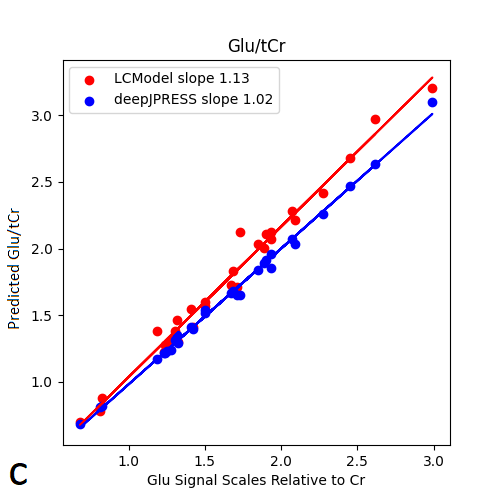

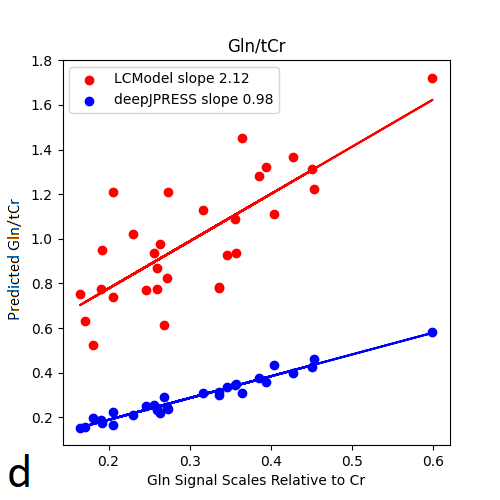


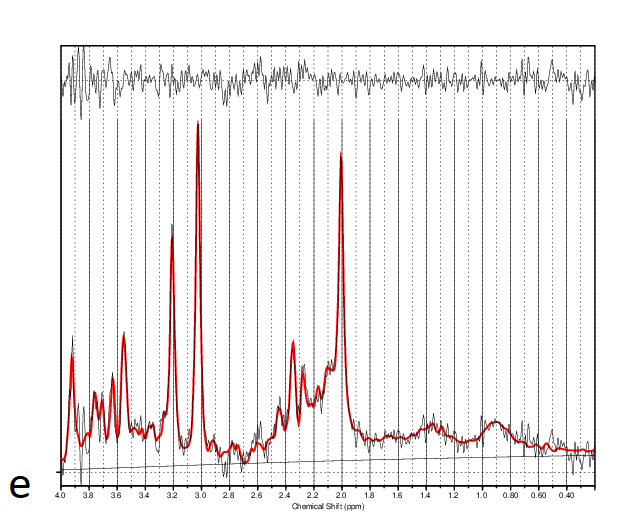


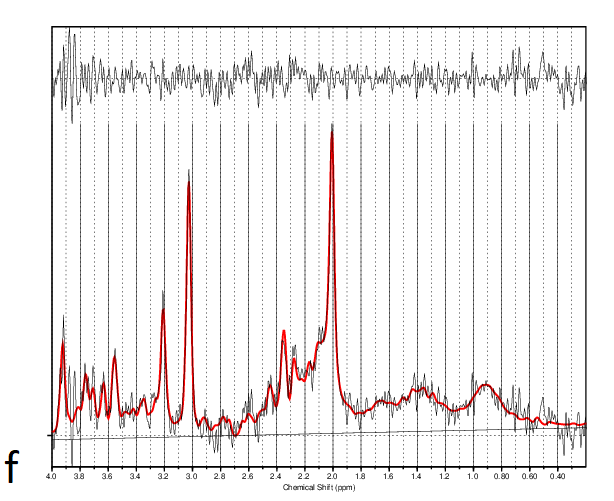


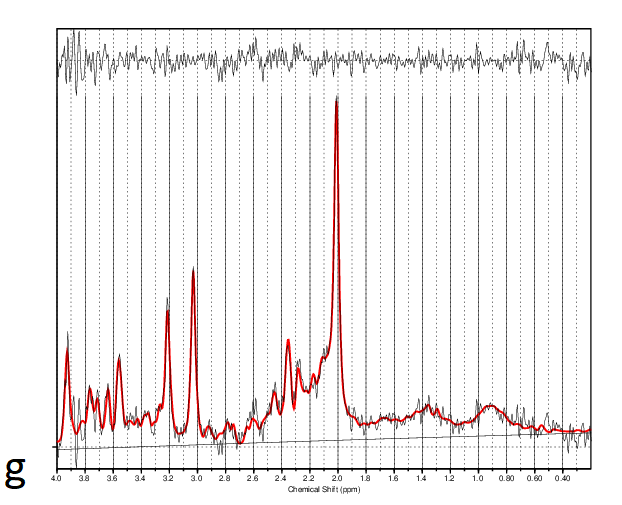


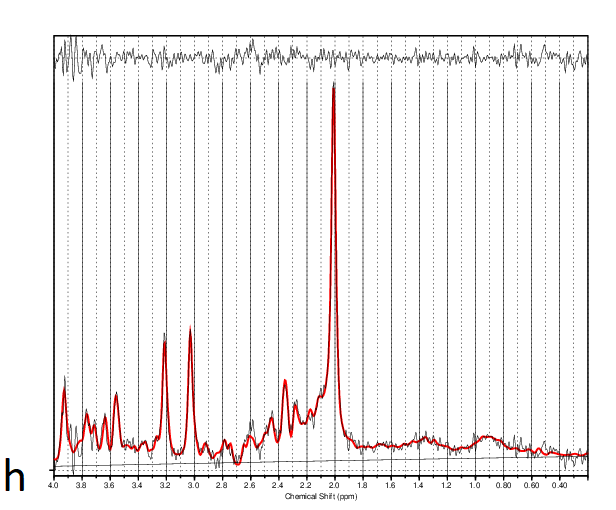


**Figure S7**: The same original in vivo dataset used in Figure S6 was employed to evaluate the effects of line broadening. Ten datasets were generated by incrementally broadening the spectral linewidth by 0 to 4.5 Hz. To match the digital resolution of LCModel, both the JPRESS input data and deepJPRESS predictions were zero-filled to 8192 points. LCModel fitting was conducted using its default settings and the vendor-provided macromolecule basis sets. The estimated tNAA concentrations and their ratios to tCr are shown in panels a and b, respectively. Four representative LCModel fits with the linewidth broadened by 0, 1.5 Hz, 3 Hz, and 4.5 Hz are presented in the left columns of panels c, d, e, and f, respectively, with the corresponding first-TE deepJPRESS predictions shown on the right. For LCModel fitting, tNAA estimates decreased linearly with increasing linewidths (a), and the fitted baselines became more oscillatory as the overall spectra became smoother (c–f, left panels) although all test datasets were derived from a single in vivo dataset. In contrast, deepJPRESS demonstrated very high robustness with respect to linewidth variations: the predicted concentration remained nearly constant (a, b), and the predicted background preserved its overall shape while showing the expected behavior of becoming smoother with increasing linewidth (c-f, right panels).

**
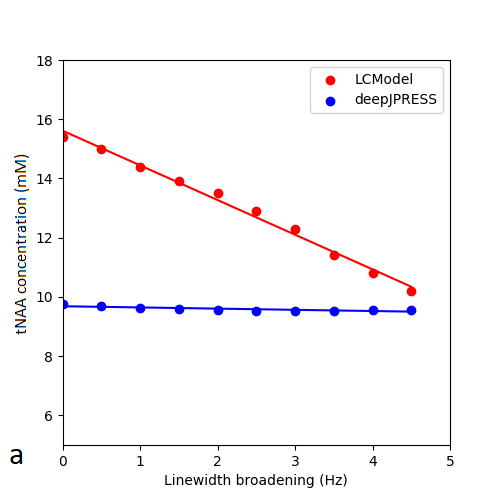

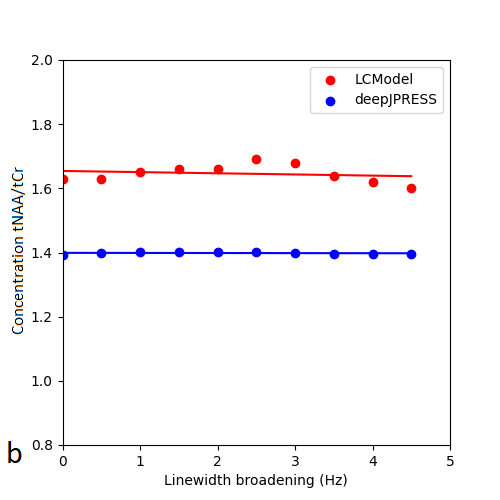
**


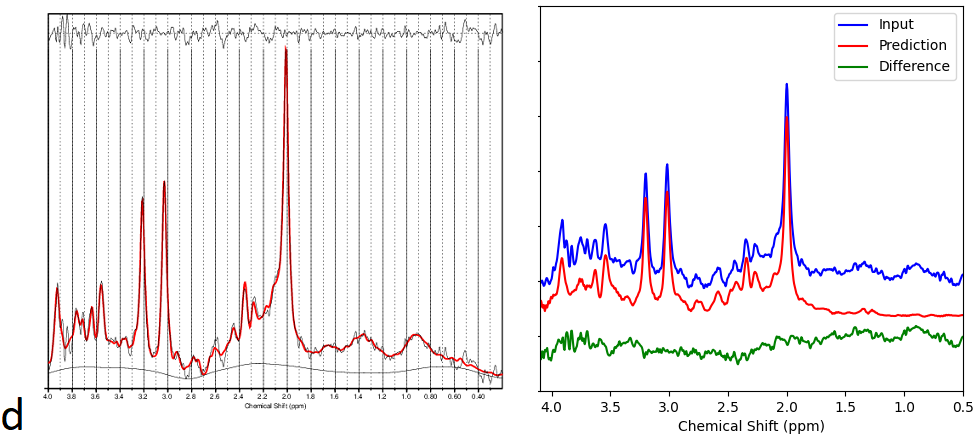


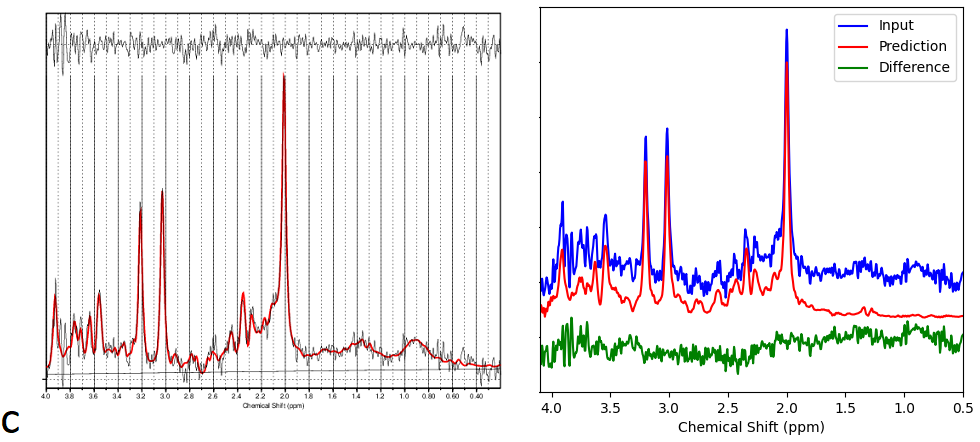


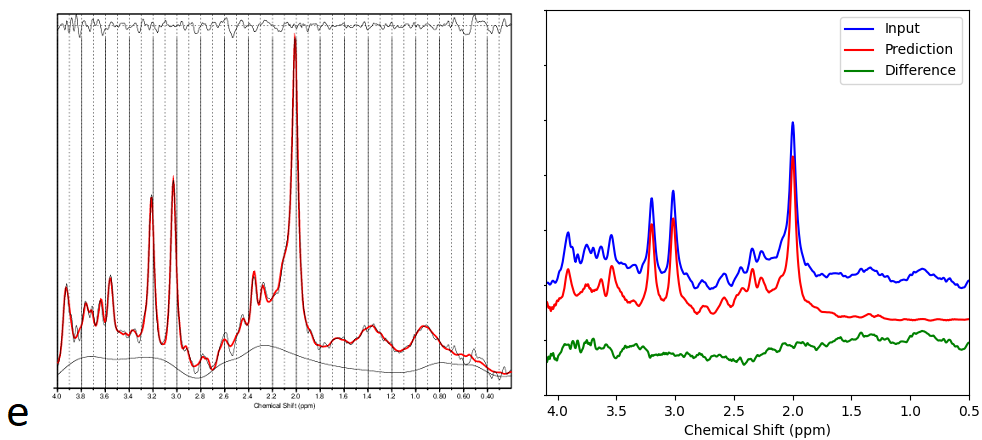

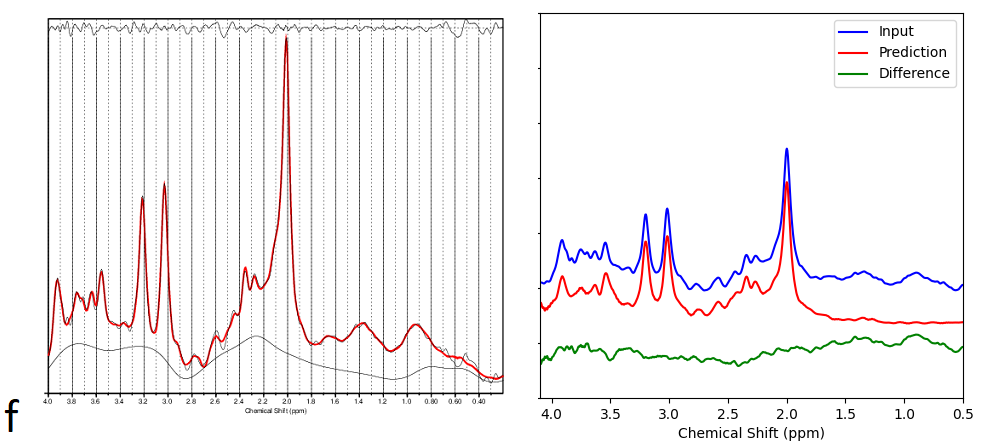

Supplement: Supplementary file 1 — Data S1: Supporting Information. [file MRM-95-3052-s001.docx]
